# A ratiometric catalog of protein isoform shifts in the cardiac fetal gene program

**DOI:** 10.1172/jci.insight.184309

**Published:** 2025-08-07

**Authors:** Yu Han, Shaonil Binti, Sara A. Wennersten, Boomathi Pandi, Dominic C.M. Ng, Edward Lau, Maggie P.Y. Lam

**Affiliations:** 1 Department of Medicine,; 2 Consortium for Fibrosis Research and Translation, and; 3 Department of Biochemistry and Molecular Genetics, University of Colorado School of Medicine, Aurora, Colorado, USA.

**Keywords:** Aging, Cardiology, Bioinformatics, Proteomics, RNA processing

## Abstract

Pathological cardiac remodeling is associated with the reactivation of fetal genes, yet the extent of the heart’s fetal gene program and its impact on proteome compositions remain incompletely understood. Here, using a proteome-wide protein ratio quantification strategy with mass spectrometry, we identified pervasive isoform usage shifts in fetal and postnatal mouse hearts, involving 145 pairs of highly homologous paralogs and alternative splicing–derived isoform proteins. Proteome-wide ratio comparisons readily rediscovered hallmark fetal gene signatures in muscle contraction and glucose metabolism pathways, while revealing what we believe to be previously undescribed isoform usage in mitochondrial and gene-expression-regulating proteins, including PPA1/PPA2, ANT1/ANT2, and PCBP1/PCBP2 switches. Paralogs with differential fetal usage tend to be evolutionarily recent, consistent with functional diversification. Alternative splicing adds another rich source of fetal isoform usage differences, involving PKM M1/M2, GLS1 KGA/GAC, PDLIM5 long/short, and other spliceoforms. When comparing absolute protein proportions, we observed a partial reversion toward fetal gene usage in pathological hearts. In summary, we present a ratiometric catalog of paralogs and spliceoform pairs in the cardiac fetal gene program. More generally, the results demonstrate the potential of applying the proteome-wide ratio test concept to discover new regulatory modalities beyond differential gene expression.

## Abstract 2

Gene isoforms have similar yet distinct functions. Using proteomics, we assessed the stoichiometric ratios of gene paralogs and splice isoforms in developing and aging hearts.

## Introduction

The proteome undergoes tremendous transformations during development as it adapts to each organ’s changing energetic and physiologic needs. In the heart, a sudden shift occurs around birth. The fetal heart switches from mainly oxidizing carbohydrates as fuel in a relatively hypoxic environment, toward preferring fatty acids under high arterial oxygen levels in the postnatal niche. This is paralleled by other development and maturation milestones, such as increased contractile forces, decreased resilience to hypoxia, and the postnatal loss of regenerative capacity (1, 2). At the molecular level, these seismic changes are underpinned by a switch in the usage of multiple fetal variants of metabolic and contractile genes toward their postnatal counterparts (3–8). Under pathological remodeling in hypertrophic and failing adult hearts, a remarkable reversal back to fetal gene expression has been widely observed (9–14). While it remains debated whether fetal gene reactivation is primarily adaptive (13, 15) or maladaptive (10) in nature, the fetal gene program has received broad interest for understanding key processes in cardiac development and disease. Many studies have successfully harnessed large-scale transcriptomics technologies to identify the trajectory of gene expression among thousands of cardiac genes and across multiple stages of fetal development (16, 17). However, the trajectory of RNA levels provides only a modest prediction of protein abundance (18–21) and cannot survey the effects of posttranscriptional and posttranslational regulations on gene expression. Therefore, discovering the extent of cardiac fetal genes at the protein level is an important goal in the study of development and disease (22, 23).

Beyond the expression of individual genes, the fetal gene program of the heart has often been investigated by comparing the relative levels within pairs of proteins. An example is the reciprocal intensity of the ɑ versus β myosin heavy chain (3, 24), which bind to other proteins to form the sarcomere structure in different chambers of the heart. Other classic examples such as actin, BCL-2, and estrogen receptors have also shown that the ratio between 2 gene products can serve as the critical determinant of their biological function rather than the individual expression of either gene (25–28). Mechanistically, this relationship can occur if 2 proteins participate in the formation of heteromultimeric complexes under stoichiometric constraints, engage in mutually repressive feedback loops, compete for the binding of the same pool of substrates, or recognize identical genetic elements (29–32). In the example of apoptotic regulators BAX and BCL-2, these 2 proteins form a heterodimer that together modulates the function of BAX, such that cells sensitized to apoptosis when BAX/BCL-2 ratio is high, whereas resistance is conferred when BAX/BCL-2 ratio is low. In another example, the ratio of the Sp family transcription factors Sp1 and Sp3 regulates many biological processes in part because they target identical promoters (33, 34). Critically, a recent large-scale analysis of genomics data has identified thousands of protein ratio quantitative trait loci (rQTL), i.e., polymorphisms that regulate protein ratios across individuals, including many rQTL that do not overlap with known individual protein level QTL (pQTL) (35). This important finding underscores that the ratio in the abundance of 2 proteins could be specifically regulated, including through shared genetic variance. Together, multiple lines of evidence indicate that the ratiometric changes in protein pairs may present an important dimension to understanding fetal gene usage, one that has a tradition rooted in classic physiology but so far has not been broadly applied to unbiased discovery.

Mass spectrometry–based proteomics can measure the levels of thousands of proteins in cardiac samples (36–39). However, typical proteomics studies aim only to compare the relative abundance of each protein across conditions, and do not explicitly test for changes in ratios between proteins. One approach to produce an unbiased catalog of protein ratios would be to exhaustively test for all pairwise proteins within a proteomics dataset, but such an all-to-all ratio comparison would involve performing many statistical tests that could inflate family-wise error rates (e.g., a list of 2,000 proteins would require making ~2 million comparisons). Thus, we choose to focus our attention on proteins that are most likely to share biologically meaningful regulations by prioritizing the comparison of isoforms, a term broadly defined to encompass members within a gene family that share considerable sequence homology, often with overlapping functions and regulatory modalities. New proteins and isoforms can arise from (i) 2 distinct genes that have diversified from gene or genome duplication events in the past, i.e., paralogs (40); or (ii) variants within a single gene that arise from exon addition or omission, i.e., differential alternative splicing (41, 42). These 2 mechanisms provide major substrates for functional diversification across cell and tissue types during metazoan evolution (40, 43), and the expression of paralogs and splice isoforms are known to be sensitive to developmental stages (44, 45).

In this study, we describe a workflow to discover statistically significant protein isoform changes from large-scale proteomics experiments, and apply it to characterize the fetal gene program of the heart. The workflow involves first acquiring absolute protein abundance estimates from quantitative mass spectrometry data, including those that utilize MS2-level tandem mass tag (TMT) multiplexing to compare multiple samples within a single experiment, followed by calculation of log abundance ratios between isoform pairs, and statistical comparisons across samples. Our analysis recapitulates hallmark fetal heart genes in sarcomeric and glycolytic pathways, while identifying potentially new candidates for fetal gene isoforms, including a switch in paralog usage along mitochondrial and gene expression regulatory pathways. At the same time, alternative splicing–derived protein isoforms (spliceoforms) provide a rich parallel source of the fetal gene program. Taken together, this study presents a catalog of the major detectable protein isoform changes in the fetal gene program of the mouse heart. We foresee this resource will complement individual gene and protein level investigations and provide insights in the studies of cardiac development, disease, and regeneration.

## Results

### Quantification of protein isoform ratios in fetal and perinatal hearts

**Figure 1:**
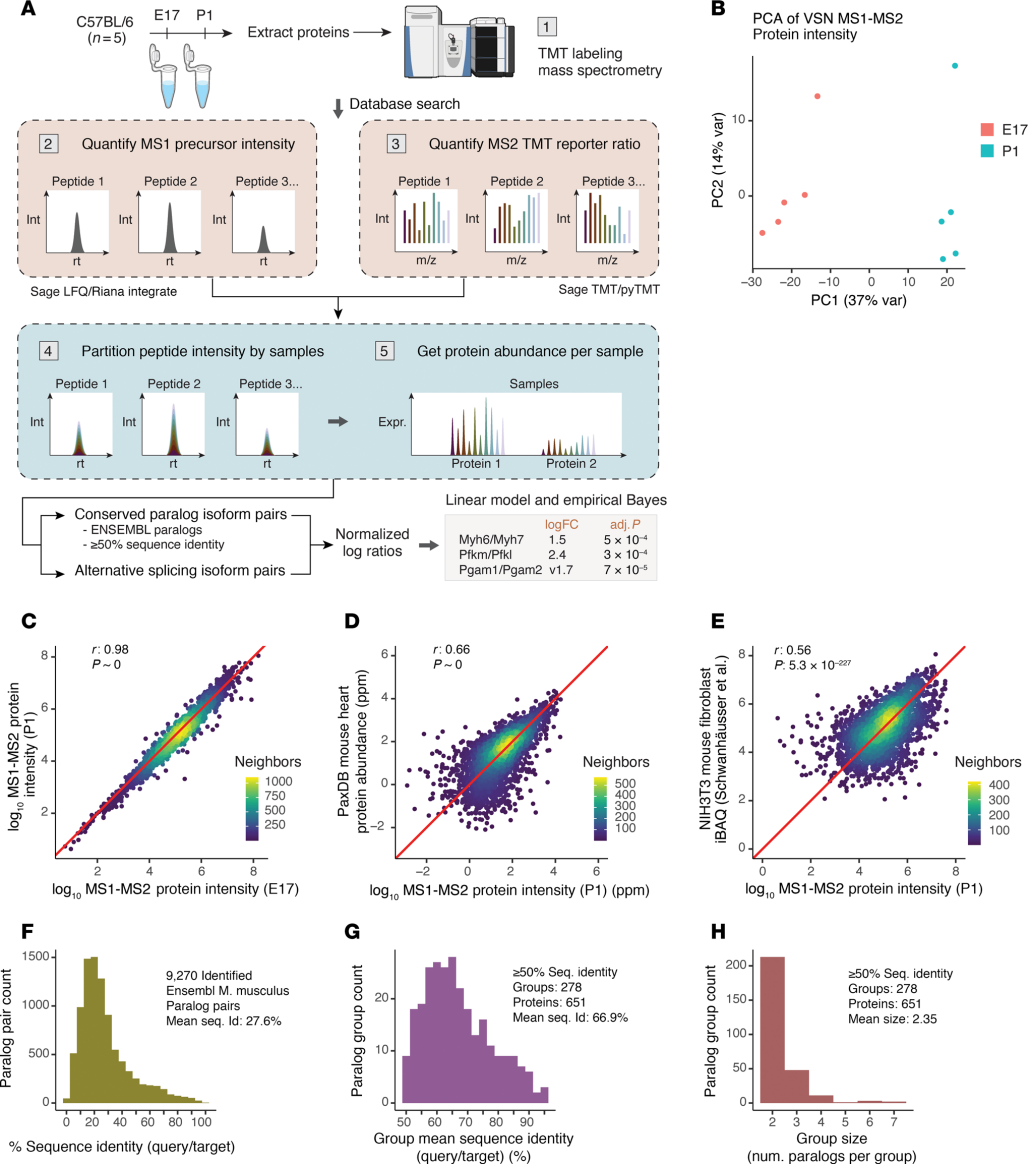
Experimental and analytical workflow of isoform usage tests. (**A**) Workflow to retrieve protein absolute quantity from TMT data and test for normalized log ratio differences. (**B**) PCA from MS1-MS2–based protein absolute quantity segregates fetal and postnatal hearts. VSN, limma:normalizeVSN() function. (**C**) The derived protein absolute quantities span more than 5 orders of magnitude and are strongly correlated between E17 and P1 samples (Pearson’s *r* = 0.98, *P* ≈ 0 in log-log scale). Color: point density. (**D**) Correlation of protein absolute quantities with known protein abundance values in the mouse heart curated from public datasets in PaxDB (Pearson’s *r* = 0.66, *P* ≈ 0 in log-log scale). (**E**) Correlation of protein absolute abundance values with mouse NIH3T3 fibroblast data in Schwanhäusser et al. (89) (Pearson’s *r* = 0.56, *P* = 5.3 × 10^–227^ in log-log scale). (**F**) Histogram showing the distribution of sequence identity (query/target) of 9,270 Ensembl-annotated *M*. *musculus* paralog pairs identified in the experiment (mean: 27.6%). (**G**) Distribution of mean sequence identity of paralog pairs in paralog groups selected for differential expression ratio analysis (≥50% sequence identity) (mean: 66.9%). (**H**) Mean paralog group size among paralog pairs selected for differential ratio analysis (mean: 2.35).

Measuring the ratio between 2 proteins requires quantifying their abundance on the same scale, i.e., estimates of absolute abundance. However, a substantial portion of proteomics studies are performed using TMT or similar MS2-based stable isotope multiplexing. We, therefore, implemented a proteomic ratio quantification workflow compatible with MS2-multiplexed mass spectrometry data (see Supplemental Methods; supplemental material available online with this article; https://doi.org/10.1172/jci.insight.184309DS1) (Figure 1A). We then generated TMT-labeled proteomics data from C57BL/6 fetal (E17) and postnatal (P1) hearts (*n* = 5 each) and applied the workflow to acquire protein absolute quantification values from the composite MS1-MS2 data (Supplemental Table 1). The resulting protein quantities clearly distinguish fetal and postnatal protein expression profiles in principal component analysis (PCA) (Figure 1B), and show good overall agreement across samples over 5 orders of magnitude (Figure 1C), demonstrating we are able to quantify both high- and low-abundance proteins consistently. To validate our quantification, we compared the derived protein absolute quantities to values from multiple mouse heart studies aggregated in PaxDB (46) (Figure 1D). The reliability of the protein quantity estimates is also reflected by a robust correlation with estimated protein copy numbers in mouse fibroblasts from an independent study, despite differences in cell type and quantification methodology (Figure 1E). These analyses indicate the absolute protein abundance estimates are accurate and not negatively affected by MS2 multiplexing.

We next gathered paralog groups within closely related gene families that were identified within the dataset, by retrieving conserved gene paralog pairs using Ensembl genome annotation. We filtered paralogs for 50% or greater sequence identity, a conservative threshold chosen based on conventional cutoffs for average sequence homologies within a protein family while excluding more distant superfamilies (47). In total, 9,270 paralog pairs were identified (Figure 1F). After filtering for sequence homology, the paralog groups consisted of 545 quantifiable pairwise paralog relationships, with an average sequence identity of 66.9% (Figure 1G). Each paralog group contained an average of 2.3 proteins (Figure 1H). We then considered the pairwise generalized log ratios of absolute quantities between paralogs within each paralog group. Diagnostic plots confirmed that the log ratios conform to limma assumptions of normality and linearity (Supplemental Figures 1 and 2). A moderated *t* test as implemented in limma was then performed on the abundance ratios of the 520 pairs of paralogs between fetal and postnatal mouse hearts (Supplemental Table 2), out of which 142 were significantly different at a conservative threshold of a limma FDR-adjusted *P* value of less than 0.01 and absolute log(fold change) of 0.5 or greater (Supplemental Table 3). To remove protein pairs where one isoform may be present only at miniscule levels, we also implemented a magnitude cutoff, a minor isoform fraction (MIF) of 0.05 or greater, yielding a final set of 125 significant paralog pairs.

We asked whether significant ratio differences cover proteins that are not differentially expressed at individual protein levels. On an individual protein level, we quantified the relative fetal/postnatal abundance of 3,889 canonical UniProt proteins, of which 1,025 were differentially expressed under identical significance thresholds (limma FDR-adjusted *P* value < 0.01 and absolute log[fold-change] ≥ 0.5) (Supplemental Table 4). The differentially expressed proteins were enriched in processes important for cardiac development, including cell cycle, mRNA splicing, mitochondrial translation, and complex I biogenesis terms (gene set enrichment analysis [GSEA] FDR < 0.01), indicating the data capture rich developmental stage–specific differences in the proteomic landscape of fetal and postnatal hearts (Supplemental Figure 2). Notably, out of 520 quantified paralog pairs, 115 pairs contained at least one protein that was not differentially expressed in single-protein comparison and 24 pairs had neither protein differentially expressed in single-protein comparison (Supplemental Table 5). Hence, the paralog ratio comparisons are able to uncover additional information under uniform significance thresholds.

### Ratio comparisons capture known paralog usage in prenatal to postnatal shift

**Figure 2:**
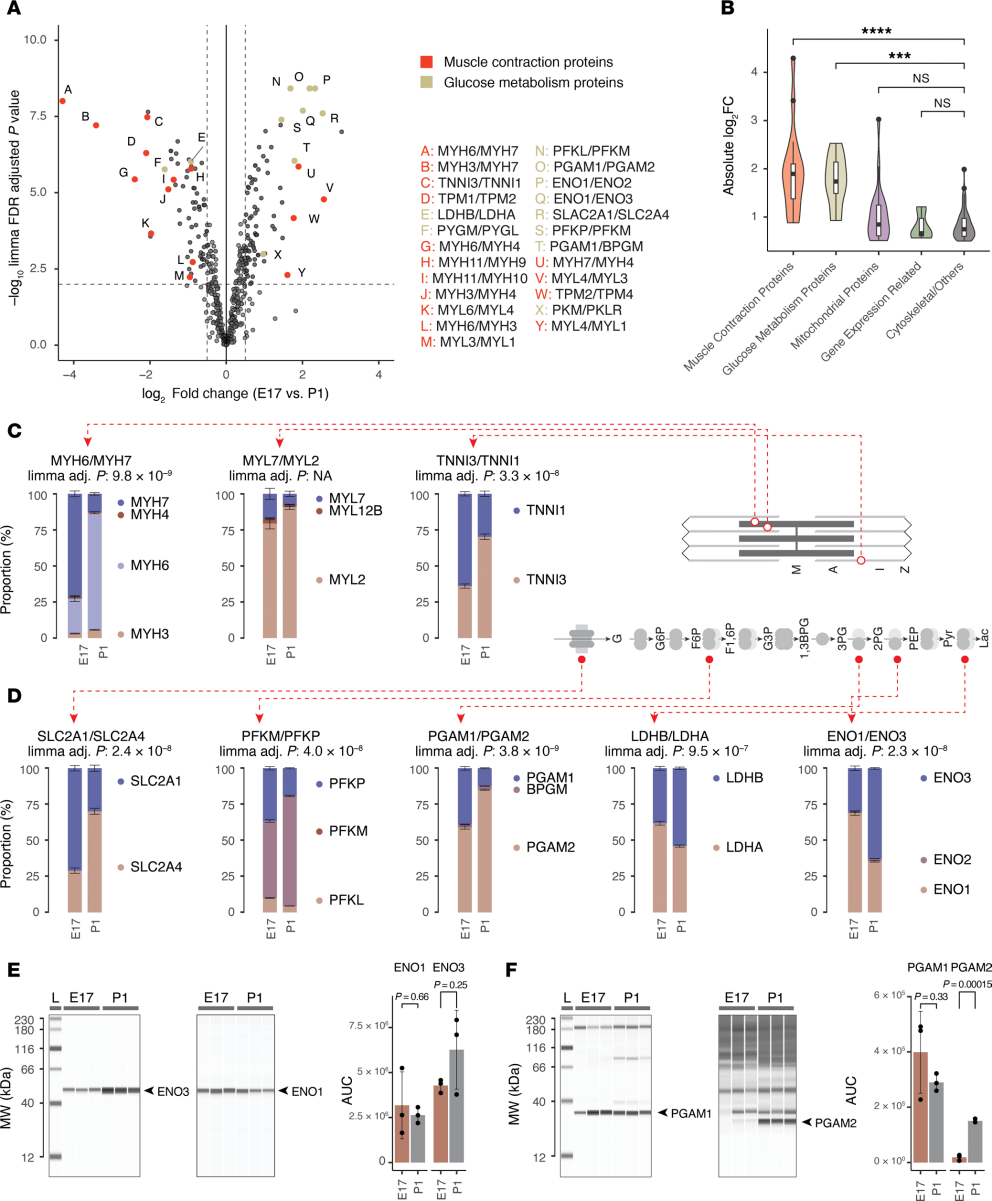
Protein isoform ratios capture hallmark fetal heart genes. (**A**) Scatterplot of log_2_(fold change) (*x* axis) versus –log_10_(*P* value) (*y* axis) of compared paralog pairs (*n* = 5 each for fetal and postnatal hearts) highlighting the significantly differentially expressed paralogs in contraction proteins (red) and glucose metabolism proteins (gold). Letters refer to paralog pairs in legends. (**B**) Violin/box plot of the magnitude of differential usage (absolute log_2_ ratio) of paralogs in different major functional categories between fetal and postnatal hearts. ***P < 0.005; ****P < 0.001 compared with “Cytoskeletal/Other” by 2-tailed *t* test. NS, *P* ≥ 0.05. FC, fold change. (**C**) Proportional bar charts showing the proportion of protein molecules in each paralog group, reflecting the known shifts in isoform usage among sarcomere genes in the fetal gene program, including (from left to right) MYH6/MYH7, MYL7/MYL2, and TNNI3/TNNI1. Error bars show SEM of cumulative proportion. *n* = 5 fetal and postnatal hearts. Paralog pairs with limma FDR-adjusted *P* < 0.01, absolute log(fold change) ≥ 0.5, average MIF ≥ 0.05 are shown, except for MYL7/MYL2, which was individually inspected as a known fetal gene program protein. (**D**) Same as **C**, but for hallmark fetal genes in glucose metabolism, from left to right: SLC2A1/SLC2A4 (GLUT1/GLUT4), PFKM/PFKP, PGAM1/PGAM2, LDHB/LDHA, and ENO1/ENO3. Paralog pairs are shown with limma FDR-adjusted *P* < 0.01 and average MIF ≥ 0.05. (**E**) Capillary-based immunoassay corroborates the relative shift from ENO1 to ENO3 in postnatal hearts. *P* values derived from 2-tailed *t* test. Error bars show SD. (**F**) Same as **E**, but for the postnatal shift from PGAM1 to PGAM2.

At E17, the fetal mouse heart has undergone morphogenetic development, including chamber differentiation, valve formation, and cardiomyocyte expansion, and morphologically resembles postnatal hearts. At the same time, cardiac maturation continues to proceed, including a gradual shift toward oxidative phosphorylation, while the retention of regenerative capacity distinguishes it from postnatal and adult hearts. Gene usage differences between E17 and P1 should therefore represent components of the fetal heart program that are relevant to postnatal maturation and adaptation. The paralog ratios recapitulate known features of the fetal genetic program, including contractile proteins and glucose metabolism proteins that show prominent isoform usage differences (Figure 2, A and B).

#### Changes in contractile protein isoform usage.

Myosin heavy chain α (MYH6) and β (MYH7) bind with actin-tropomyosin complexes in the sarcomere of different muscle fiber types. In mice, MYH7 is a well-established fetal gene (7, 24). Consistently, the data recapitulate a prominent, approximately 20-fold reciprocal shift from the β to α form from E17 to P1 hearts; at E17, the MYH7/MYH6 ratio was >2 to 1, whereas in P1 hearts MYH6 was more than 8-fold higher (Figure 2C). The developmental stage–specific expression of fetal troponin I is another hallmark in fetal development (4, 48). Likewise, the data revealed a greater than 4-fold relative shift from slow skeletal muscle type troponin I (TNNI1) toward the adult cardiac type (TNNI3), where they were expressed approximately 1:1 at E17 but TNNI3 was greater than 4-fold higher in P1 hearts (Figure 2C). Other changes included a greater than 5-fold further enrichment of the ventricular MYL3 over fetal/essential MYL4 in postnatal hearts, and a 2.6-fold shift from the non-muscle myosin polypeptide MYH10 toward the smooth muscle form MYH11, where MYH10 was 2-fold higher than MYH11 in fetal heart, but lower in postnatal hearts (Supplemental Table 3).

#### Changes in glucose metabolism protein isoform usage.

Prominent changes were also seen among proteins that function in glucose metabolism. We consider here 5 known fetal heart genes. (i) First, fetal hearts expressed more basal glucose transporter GLUT1 (SLC2A1) than insulin-sensitive GLUT4 (SLC2A4) at an approximately 70%:30% ratio, but roughly opposite proportions postnatally (30%:70%) (Figure 2D). A transition from GLUT1 to GLUT4 in neonatal development is known in rodent hearts and brown fat (49), whereas prior work showed both GLUT1 and GLUT4 transcripts are more highly expressed in adult than fetal human hearts (15). (ii) Phosphofructokinase-1 (PFK-1; EC 2.7.1.11) is a critical glycolytic enzyme that catalyzes the committed step of fructose 6-phosphate to fructose 1,6-bis phosphate. PFK-1 has 3 isozymes encoded by 3 paralog genes: PFK-M (muscle), PFK-P (platelet), and PFK-L (liver), with adult hearts expressing PFK-M exclusively. The ratio proteomics data recapitulated an expansion of PFK-M from fetal heart (53%) to postnatal heart (76%) coupled to the contraction of PFK-L (10% to 4%) and PFK-P (37% to 19%) isoforms (Figure 2D), consistent with isozyme biochemical activity assays (50). (iii) In another glycolytic enzyme, phosphoglycerate mutase (PGAM1/2; EC 5.4.2.11), the proportion of the skeletal muscle and myocardial enriched type-M isoform (PGAM2) increased from 59% to 85% between fetal to postnatal hearts, whereas the proportion of the type-B isoform PGAM1 decreased from 39% to 12% (Figure 2D). (iv) In fetal hearts, α enolase (ENO1; EC 4.2.1.11) was the predominant isoform, amounting to 69% of abundance within the paralog group, but decreased to 35% in postnatal heart (Figure 2D). This was coupled with an increase from 30% to 63% of the striated muscle isoform of enolase (β enolase, ENO3), an approximately 4-fold switch in ENO1/ENO3 ratios. (v) In fetal hearts, lactate dehydrogenase (EC 1.1.1.27) H and M subunits (LDHA)/(LDHB) existed at a 62%:38% ratio, versus 46%:54% in postnatal hearts, a 1.9-fold switch (Figure 2D). This shift is consistent with the known enrichment of LDH complexes containing the H subunit LDHB in the heart and its preference for conversion of lactate to pyruvate in oxidative metabolic environments, in contrast with the preference of the M subunit to favor lactate production from pyruvate in glycolytic tissues (51). Other examples include a decrease in the proportion of non–muscle-type cytosolic creatine kinase (CKB), but a reciprocal increase in the proportion of mitochondrial matrix creatine kinase (CKMT2) (Supplemental Table 3).

Thus, the proteome ratio test captures well-established changes in sarcomeric and glycolytic isoform usage.

#### Validation by orthogonal experiments and additional proteomics data.

To validate the isoform ratios, we adopt 2 orthogonal approaches. First, we note that although immunoblots can measure the relative fold change of a protein between conditions, they cannot directly quantify the absolute proportion of 2 proteins without extensive calibrations to map measurements to absolute concentration, due to the different affinity of antibodies for epitopes. Antibodies may also conflate closely related sequences. Nevertheless, capillary-based immunoassays (ProteinSimple Jess) using separate antibodies against ENO1 and ENO3 show a consistent postnatal toward ENO3 relative to ENO1 on E17 versus P1 (Figure 2E). Likewise, capillary-based immunoassays show a suggestive decrease in PGAM1 band intensity and a significant increase in PGAM2 band intensity, in agreement with the ratio proteomics data (Figure 2F).

**Figure 3:**
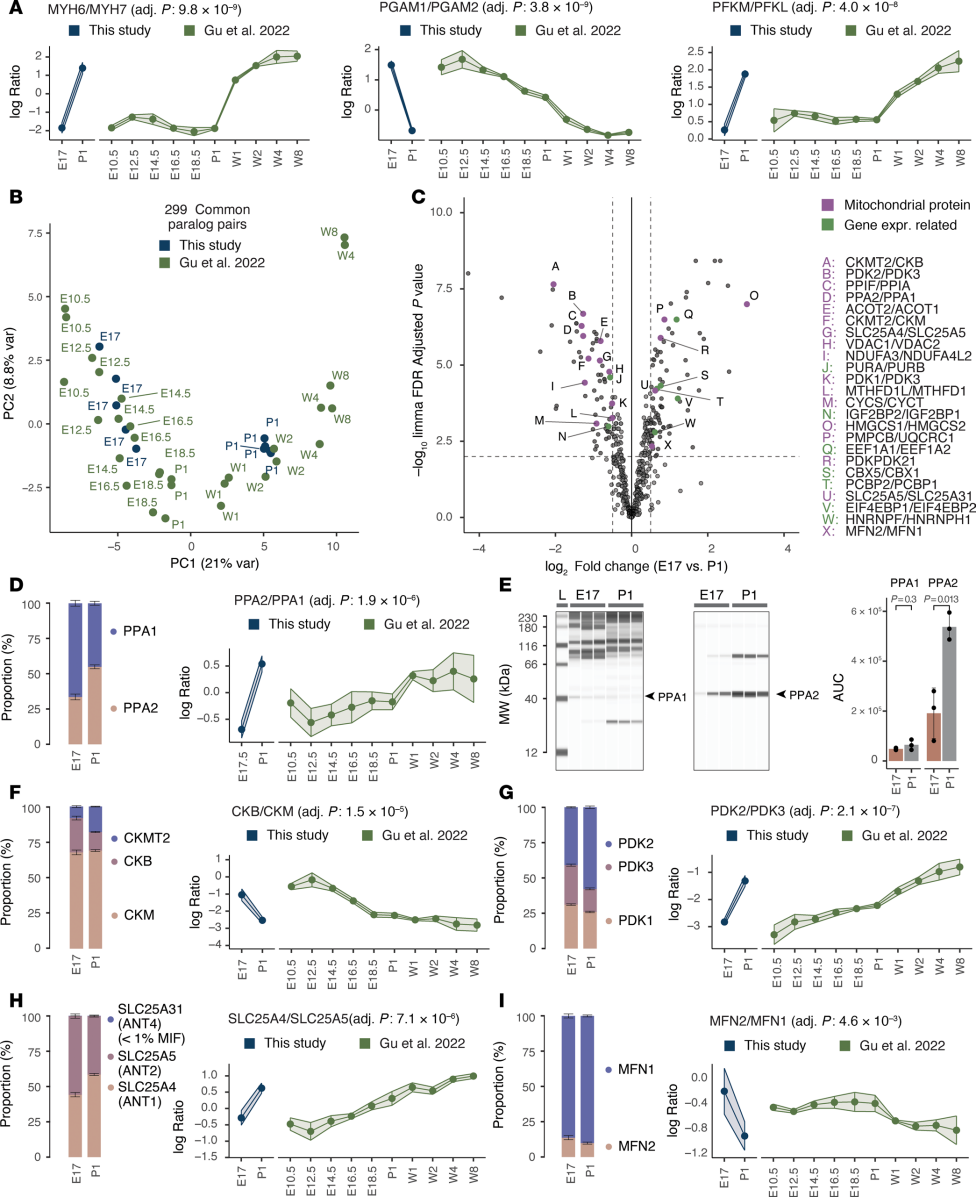
Detection and validation of paralog usage shifts in mitochondrial proteins. (**A**) Application of ratiometric calculation to prior data recapitulated the rise in MYH6/MYH7, PGAM2/PGAM1, and PFKM/PFKL ratios in postnatal hearts across datasets. Adj. *P*, limma FDR-adjusted *P* value in E17 versus P1 (*n* = 5) in this study. Error bars and ribbon width show SD. (**B**) PCA of the abundance ratios of 299 commonly quantified paralog pairs distinguished developmental stages in the current study and reanalysis of an existing dataset of pre- and postnatal C57BL/6 mouse hearts from Gu et al. (23). (**C**) Scatterplot of log_2_(fold change) (*x* axis) versus –log_10_(*P* value) (*y* axis) of compared paralog pairs (*n* = 5 each for E17 and P1 hearts) highlighting the significantly differentially expressed paralogs in mitochondrial proteins (purple) and gene expression regulation–related proteins (green). Letters refer to paralog pairs in legends. (**D**) Proportional bar chart (left) showing an increase in the PPA2/PPA1 ratio from E17 to P1 mouse heart (limma FDR-adjusted *P* = 1.9 × 10^–6^), consistent with the postnatal increase in the PPA2/PPA1 ratio in the reanalysis of Gu et al. (right). (**E**) Capillary-based immunoassay corroborates the relative shift from PPA1 to PPA2 in postnatal hearts. *P* values derived from 2-tailed *t* test. Error bars show SD. (**F**) Postnatal isoform usage shifts in CKB/CKMT across the 2 datasets. Left: Bar chart of cumulative proportion. Error bars show SEM. Right: Ribbon chart. Error bars and ribbon width show SD. (**G**) Same as **F**, but for PDK2/PDK3. (**H**) Same as **F**, but for SLC25A4/SLC25A5. (**I**) Same as **F**, but for MFN1/MFN2.

As a second approach to corroborate our findings, we applied our MS1-MS2 ratiometric calculation and statistical testing workflow to public TMT-labeled proteomics data on embryonic (E10.5–E18.5) and postnatal (P1 to postnatal week 8) C57BL/6 mouse hearts (23). The analysis again reproduced hallmark sarcomeric and glycolytic fetal heart proteins, underscoring the consistency of ratio calculations across independent datasets (Figure 3A). Moreover, the isoform ratios of 299 common paralog pairs shared across datasets readily segregated pre- and postnatal samples across time points (Figure 3B). Thus, we show that paralog usage alone is sufficient to distinguish cardiac developmental stages, and proteomics ratio tests present a powerful method to capture hallmark fetal genes at the protein level.

### Ratio comparisons identify candidate isoform shifts in fetal heart development

Our analysis revealed potential new candidates of the fetal gene program, many of which function in mitochondrial and gene expression regulation pathways (Figure 3C).

#### Mitochondrial protein isoform rewiring.

We found changes in mitochondrial proteins, including some not formally associated with the fetal gene program of the heart, including (i) a 2.4-fold reciprocal swing from inorganic diphosphatase PPA (EC 3.6.1.1) cytoplasmic isoform 1 (PPA1) to the mitochondrial isoform 2 (PPA2) (limma FDR-adjusted *P* = 1.9 × 10^–6^), from 66%:34% to 45%:55% (Figure 3D). This postnatal increase in PPA2 was verified by our reanalysis of existing proteomics data (Figure 3D) and experimentally by capillary-based immunoassay (Figure 3E). (ii) Relative shift from CKB (EC 2.7.3.2) toward the type-M creatine kinase (CKM) polypeptide chain, in roughly 70%:25% to 70%:10% fashion (Figure 3F). The rest of the creatine kinase pool is accounted for by a postnatal increase in sarcomeric mitochondrial creatine kinase (smtCK) (CKMT2), which increased from less than 10% in fetal hearts to approximately 20% of the pool in perinatal hearts, suggesting a reconfiguration of creatine kinase isozymes to increase phosphocreatine production for contractile demands. The decrease in the CKB/CKM ratio was reaffirmed in the reanalysis of prior proteomics data.

The isoform switches cannot be fully explained by a simple increase in postnatal mitochondrial density, because wholly mitochondrial protein isoform groups also show differences in usage, suggesting changes in mitochondrial protein compositions. For instance, (iii) we observed a switch from pyruvate dehydrogenase kinase 1 and 3 (PDK1/PDK3) toward PDK2 as the major PDK isozyme (Figure 3G). (iv) In parallel, the cardiac skeletal muscle form of ADP/ATP carrier ANT1 (SLC25A4) replaced the fibroblast form (SLC25A5) ANT2 as the prevalent mitochondrial ADP/ATP carrier (Figure 3H). (v) This was accompanied by a further increase in MFN1/MFN2 ratios (Figure 3I) and (vi) a further increase in the mitochondrial voltage-dependent anion channel ratio (VDAC1/VDAC2 ratio) (Supplemental Table 3).

Other proteins relevant to mitochondrial biology include a shift in sideroflexin, a mitochondrial membrane transporter involved in heme metabolism and serine transport (52, 53), from the ubiquitous isoform SFXN1 toward the neuron-enriched SFXN3 (Supplemental Table 3). In parallel, hydroxymethylglutaryl-CoA (HMG-CoA) synthase (EC 2.3.3.10) cytosolic isoform HMGCS1 decreased, coupled to an increase in the mitochondrial form HMGCS2, leading to a strong switch toward HMGCS2 usage (log[fold change] = 3.03, limma FDR-adjusted *P* = 1.1 × 10^–7^), such that the proportion of HMGCS1 dropped from 14% of the total HMGCS pool at E17 to 2% at P1. An HMGCS1 decrease during prenatal cardiac development is known to impact the mevalonate pathway for cholesterol biosynthesis (22). The data here, in turn, reveal a parallel increase in the mitochondrial form in early postnatal hearts.

From fetal cardiac development toward birth, there is a gradual shift from glycolytic toward aerobic metabolism in the heart, parallel to mitochondrial biogenesis (54). Our analysis suggests that metabolic remodeling in postnatals hearts also involves broad changes in mitochondrial protein isoform usage in parallel with glycolysis proteins.

#### Posttranscriptional and translational gene regulation programs.

**Figure 4:**
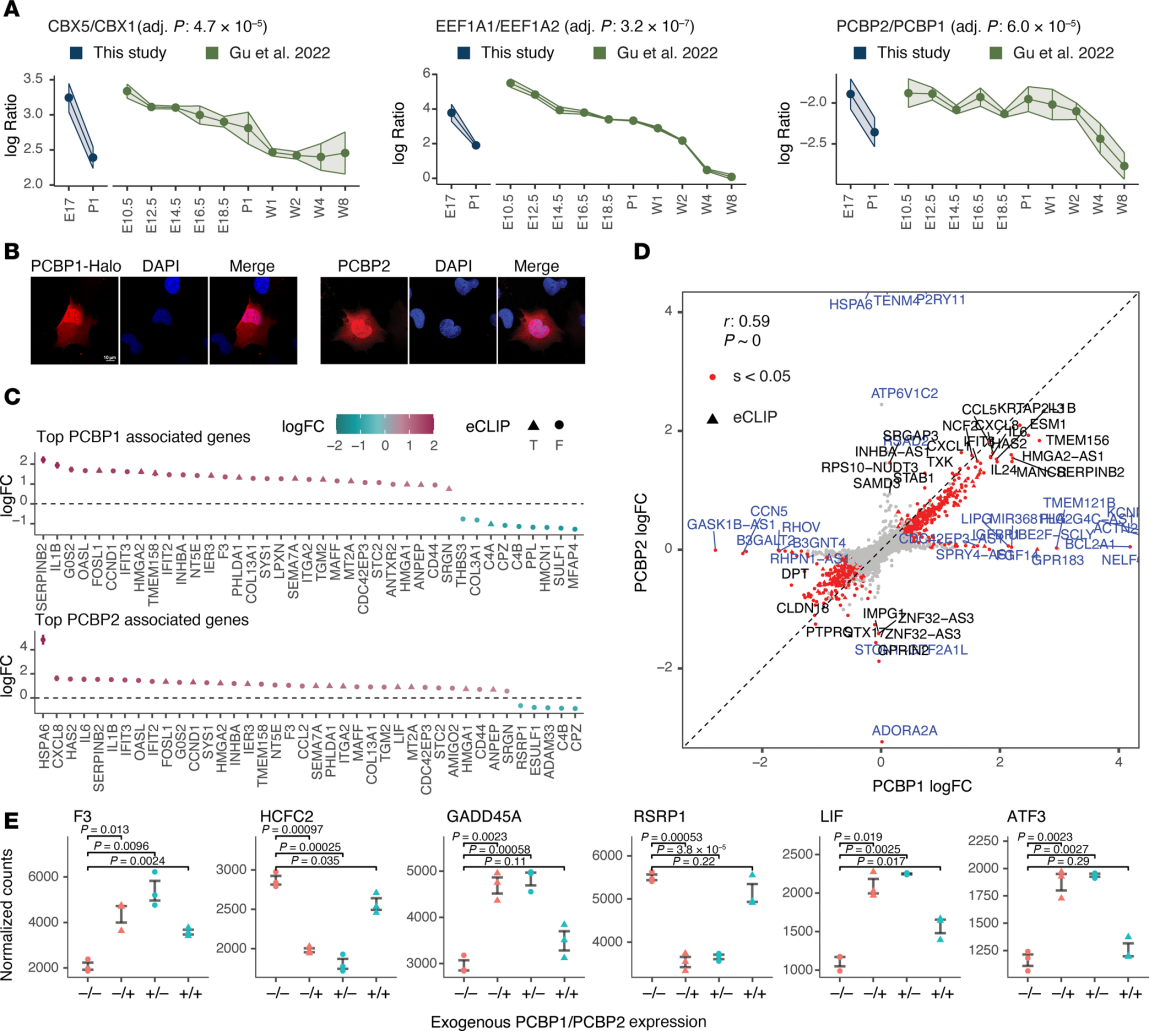
Examples of isoform usage shifts in gene regulation–related proteins. (**A**) Postnatal shifts in the ratio of gene expression regulation–related proteins in data generated in this study and in the reanalysis of Gu et al. (23) limma FDR-adjusted *P* value (Adj. *P*) in E17 versus P1 (*n* = 5) in this study. Error bars and ribbon width show SD. (**B**) Immunofluorescence images of human AC16 cardiac cells overexpressing HaloTag-linked PCBP1 and PCBP2, showing nucleocytoplasmic localization of both proteins. Scale bar: 10 μm. (**C**) RNA-sequencing data showing the top differentially expressed genes associated with the expression of exogenous PCBP1 (top) and PCBP2 (bottom) in human AC16 cells. Colors: log_2_(fold change) (logFC) versus control cells. Triangles: known binding target of PCBP1 or PCBP2 in ENCODE eCLIP data (90). (**D**) Scatterplot showing the overall similarities in the RNA abundance profiles of AC16 cells in PCBP1 (*x* axis) and PCBP2 (*y* axis) overexpression over control cells (Pearson’s *r* = 0.59, *P* ≈ 0 in log-log scale). Red data points: significantly differentially expressed genes (DESeq2 shrinkage *s* value < 0.05). Blue labels: genes that show different regulations (off-diagonal genes) between PCBP1 and PCBP2. (**E**) Dot plots showing the effects of PCBP1 and/or PCBP2 overexpression on the abundance (normalized counts) of select genes that show an interaction effect. –/–, no overexpression; –/+, PCBP1 overexpression only; +/–, PCBP2 overexpression only; +/+, concurrent PCBP1 and PCBP2 overexpression in the same cells.

Changes in this category include (i) a switch in the chromobox protein homologs CBX5/CBX1; (ii) the translation elongation factor α (EEF1A) from the “ubiquitous” EEF1A1 form toward the heart/muscle form EFF1A2, where the former is 6.5-fold higher in fetal hearts but approximately 3-fold higher in P1 hearts and decreases further in later time points; and (iii) the poly(rC)-binding proteins PCBP1 and PCBP2 (Figure 4A). To explore potential functional implications of isoform usage shifts, we examined the effect of PCBP1 and PCBP2 expression in human AC16 cardiac cells. PCBP1 and PCBP2 are KH-domain–containing RNA-binding proteins (RBPs) that have been implicated in transcriptional (55), posttranscriptional (56), and translational regulations (57). How PCBP1 and PCBP2 are functionally differentiated remains a subject of interest (56) and few reports have outlined their function in cardiac cells. The 2 paralogs share close homology (~85% identical sequences) and partially overlapping RNA binding targets, suggesting they may form mutually regulatory relationships. Human and mouse PCBP1 sequences share 100% identity, whereas human and mouse PCBP2 are identical except in 5 positions; therefore, we expressed the human/mouse PCBP1 and human PCBP2 coding sequences in human AC16 cardiac cells. Immunofluorescence imaging confirmed broad cytonuclear localization of both paralogs under overexpression (Figure 4B). mRNA sequencing of cells overexpressing PCBP1 or PCBP2 revealed the differential expression of 1,362 and 672 transcripts, respectively (shrinkage *s* value < 0.05; absolute log[fold change] > 0.25), including known eCLIP targets and other genes, suggesting the differential expression may arise from both direct RBP target regulations and secondary effects (Figure 4C). A comparison of differential gene expression between PCBP1 and PCBP2 showed a high concordance (Pearson’s *r* = 0.59 in log-log scale; *P* ≈ 0), which is consistent with the 2 proteins having overlapping roles in functional phenotypes (Figure 4D). Nevertheless, a small number of off-diagonal data points indicate the 2 paralogs show functional specialization by influencing the expression of nonoverlapping genes (Figure 4D). Notably, some differential expression events, including F4, HCFC2, GADD45A, RSRP1, LIF, and ATF3, were partially neutralized when both PCBP1 and PCBP2 were overexpressed together in the same cells (Figure 4E), which is suggestive of the notion that their differential ratio rather than individual abundance could be a determinant of cellular phenotypes.

#### Other proteins.

Isoform switches that did not fall into the above categories include a switch from DYNLL1 toward DYNLL2 as the major isoform in postnatal hearts (50%:50% in fetal hearts vs. 32%:68% in postnatal hearts); a minor switch of vimentin (VIM)/desmin (DES) ratios (76%/:24% to 64%:35%); and a minor increase in lamin B2 (LMNB2) versus B1 (LMNB1) (Supplemental Table 3). In parallel, there was an approximately 2-fold further enrichment of fatty acid binding protein FABP3 over FABP4 (from 60:40 ratios to 75:25 ratios) in postnatal hearts (Supplemental Table 3), which was also previously observed in single-cell transcripts in developing mouse hearts from E9.5 to P21 (17). Taken together, the results demonstrate widespread changes in protein isoform usage during cardiac development, including what we believe to be previously undescribed fetal gene candidates. The reliability of the protein ratio analysis is underscored by the identification of classic hallmark fetal gene program proteins, validation by external mass spectrometry datasets, and corroboration by immunoassays.

### Fetal to postnatal isoform shifts are enriched in evolutionarily recent paralogs

**Figure 5:**
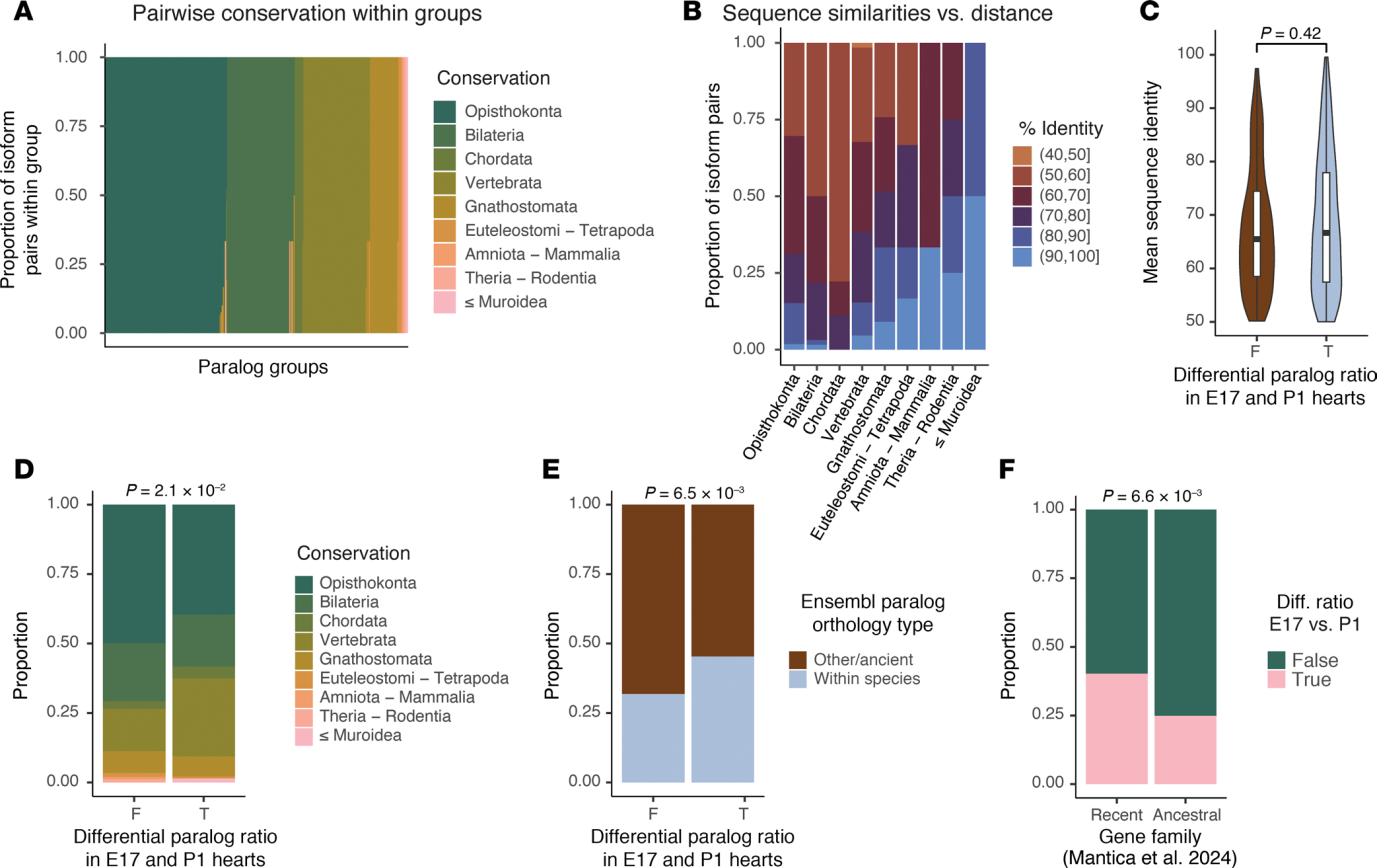
Evolutionary conservation of fetally regulated paralogs. (**A**) Sorted proportional bar charts showing the evolutionary conservation of all paralog pairs within each of the paralog groups from the most deeply conserved (across opisthokonta) to the least (Muroidea to *Mus*). (**B**) Proportional bar charts showing the binned average sequence homology among all paralog pairs within each conservation distance category. Open parenthesis “(” = noninclusive interval; closing bracket “]” = inclusive interval. (**C**) Violin/box plots of paralog differential expression status in fetal hearts versus mean sequence identity (query/target). *P* value derived from 2-tailed *t* test. (**D**) Proportional bar charts showing the ancestry distribution of paralog pairs that have consistent versus differential expression ratios in E17 versus P1 hearts. *P* values derived from Fisher’s exact test for opisthokonta membership. (**E**) Proportional bar charts showing Ensembl paralog type versus differential expression ratios in E17 versus P1 hearts. *P* values derived from Fisher’s exact test. (**F**) Proportional bar charts showing ancient versus recent gene family categorization of paralog pairs from Mantica et al. (43) against differential expression ratios in E17 versus P1 hearts. *P* values derived from Fisher’s exact test.

We next examined the relationship between isoform differential usage and evolutionary conservation. Consistent with known ancestry of gene families, the majority of quantified paralogs belong to families that are ancient (inferred to originate in or before the common ancestors of all bilaterians) (Figure 5A and Supplemental Table 6). Expectedly, more recent paralogs share greater sequence homology as defined by the mean (query/target and target/query) sequence identity following ClustalO (58) sequence alignment (Figure 5B). We noticed a modest trend for higher average sequence identity between paralogs that are differentially expressed in fetal versus postnatal hearts, although this trend was not statistically significant (2-tailed *t* test *P* = 0.42) (Figure 5C). On the other hand, we observed a statistically significant relationship showing that more ancestral paralog pairs, such as those conserved across opisthokonta lineages, are less likely to be differentially expressed in fetal versus postnatal hearts (Fisher’s exact test *P* = 0.021) (Figure 5D). Likewise, when comparing Ensembl paralog types, which distinguish ancient paralogs as those inferred indirectly from supertrees, ancient paralogs are less likely to be differentially expressed (Fisher’s exact test *P* = 6.5 × 10^–3^) (Figure 5E). To further corroborate this observation, we compared fetal expression specialization with evolutionary conservation in bilateria, using a recent phylogenetic reconstruction of gene expression profiles from 20 species to infer gene ancestry (43). By considering gene families that originated from or predated the common ancestor of bilaterians to be ancient and those within more recent families to be recent (40), the results again indicate that evolutionarily recent genes are significantly more likely to be regulated in fetal expression (Fisher’s exact test *P* = 6.6 × 10^–3^) (Figure 5F). Therefore, expression specialization in fetal hearts involves many evolutionarily recent paralogs, consistent with the notion of continued functional specialization and neofunctionalization of paralogs during metazoan evolution.

### Spliceoforms compose a second source of fetal isoform ratio shifts in the heart

Alternative splicing presents a second mechanism to generate gene isoforms. At present, the identification of splicing-derived isoform proteins (spliceoforms) remains hindered by experimental and data analytical challenges (42, 59, 60). For instance, alternative isoforms are often found in much lower abundance than their canonical counterparts. Splice junctions between exons are known to preferentially code for lysines, which become cleaved by trypsin in typical proteomics workflows, leading to the loss of exon-exon connection information in mass spectrometry experiments. Furthermore, many isoforms are tissue specific and may not be catalogued in common protein sequence databases.

**Figure 6:**
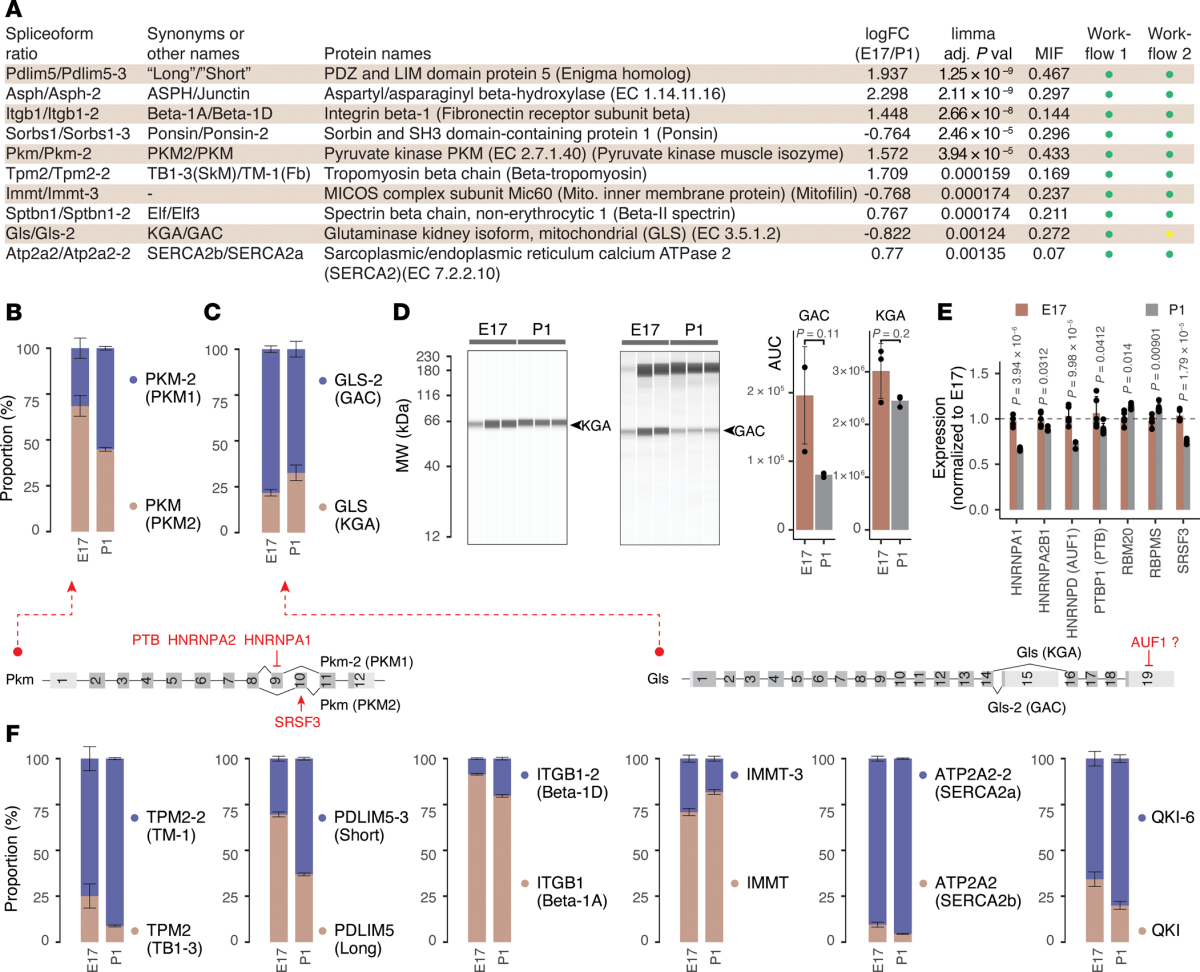
Alternative splicing–derived protein isoforms as a source of fetal genes. (**A**) Names and synonyms of 10 spliceoform pairs that are significantly different between E17 verses P1 hearts in both database search/quantification workflows. The values of limma FDR-adjusted *P* value (adj.P.Val) and log_2_(fold changes) (logFC) refer to results of workflow 1 (Sage against UniProt/Swiss-Prot isoforms; see Supplemental Methods). Workflow 2: MSFragger/Riana/pyTMT against JCAST-generated tissue-specific protein sequence database (see Supplemental Methods). Green: FDR ≤ 0.01, absolute logFC ≥ 0.5, MIF ≥ 0.05. Yellow: FDR ≤ 0.01, MIF ≥ 0.05. (**B**) Proportional bar charts showing the estimated proportions of PKM splice isoforms (M1 and M2), reflecting the known shifts from M2 to M1 in postnatal hearts. Error bars show SEM of cumulative proportion. *n* = 5 fetal and postnatal hearts. (**C**) Same as **B**, but for GLS isoforms KGA and GAC. (**D**) Capillary-based immunoassay corroborates the relative shift from GAC to KGA in postnatal hearts. *P* values derived from 2-tailed *t* test. Error bars show SD. (**E**) Bar charts showing the normalized (to E17) expression of splice factors and RNA-binding proteins implicated in isoform shifts. Numbers denote limma FDR-adjusted *P* values in E17 versus P1 hearts. (**F**) Proportional bar charts for additional splice isoform pairs TPM2 (TM-1 vs. TB1-3), PDLIM5 (long vs. short), ITGB1 (β-1D vs. β-1A), IMMT (canonical vs. TB1-3), ATP2A2 (SERCA2a vs. SERCA2b) and QKI (canonical vs. QKI-6). Error bars show SEM of cumulative proportion. *n* = 5 fetal and postnatal hearts. Selected spliceoform pairs are shown with limma FDR-adjusted *P* < 0.01 and average MIF ≥ 0.05.

To examine spliceoform ratios in fetal and postnatal hearts, we first searched the data against mouse UniProt/Swiss-Prot canonical and isoform sequences. Applying the same ratio tests as the paralog comparisons (FDR-adjusted *P* < 0.01, MIF ≥ 5%, absolute log_2_[fold change] ≥ 0.5), we found 11 isoform pairs with significant ratio differences between E17 and P1. To verify the identifications, we used an RNA-sequencing-informed identification pipeline we previously described (JCAST) (see Supplemental Methods). Among commonly identified proteins, both workflows returned highly similar protein absolute values over 5 orders of magnitude (correlation 0.85–0.86) (Supplemental Figure 4). The JCAST workflow corroborates 10 of the 11 significant isoform shifts found in our main search strategy (Figure 6A) and further uncovered 9 additional significant isoform pairs at an adjusted *P* value of less than 0.01 and MIF of greater than 0.05, bringing the total number of protein spliceoform signatures to 20 (Supplemental Table 7).

Pyruvate kinase (EC 2.7.1.40) has 2 main spliceoforms in the heart: the adult M1 form and the fetal M2 form (n.b., the M2 isoform is the designated canonical entry on UniProt and Ensembl; hence, the M1 isoform’s assigned database name here is PKM-2) (Figure 6B). The M2 isoform differs from M1 by a pair of mutually exclusive exons 9 (M1) and 10 (M2) and is known to be reactivated in failing hearts (61). Protein ratio comparison showed an expected switch of usage from M2 to M1 in P1 hearts. Glutaminase 1 (GLS1; EC 3.5.1.2) is a rate-limiting enzyme in glutaminolysis, and switches from the high-activity GAC to the KGA isoform in P1 (Figure 6C). This switch was partially corroborated by immunoassays, despite caveats of the approach as stated above (Figure 6D). Upregulation of glutaminolysis has been implicated in cardiac hypertrophy and failure (62), suggesting GLS may form a part of the constellation of fetal metabolism genes with differential usage during cardiac development and remodeling.

These differential isoform usages are underpinned by concurrent RBP regulations in postnatal hearts. The splicing of PKM2 is controlled by 3 splice factors, HNRNPA1, HNRNPA2, and PTB, which function together to suppress the inclusion of the M1-specific exon 9 (63). Another splice factor, SRSF3, promotes the exclusion of the M2-specific exon 10. Consistently, we found that these splice factors were preferentially expressed in E17 and depleted in P1, which would elevate PKM2 expression in fetal hearts (Figure 6E). The regulation of KGA and GAC usage is complex and likely involves multiple RBPs, lncRNAs, and proteolytic factors (64, 65). In particular, HNRNPD (AUF1) is known to bind AU-rich elements at AUUUA pentamers (66), which are present in the 3′ UTR of both GAC and KGA transcripts. Prior analysis of rat gene sequences suggests that AUF1 may destabilize KGA (65). In our hands, AUF1 is repressed at P1 (llimma FDR-adjusted *P* = 1 × 10^–4^) (Figure 6E), suggesting it may play a role in the postnatal isoform shift.

Mammals have 4 tropomyosin genes: TPM1, TPM2, TPM3, and TPM4. Moreover, each of the 4 has splicing-derived isoforms. Cardiac muscles predominantly express α-tropomyosin (TPM1), whereas β-tropomyosin (TPM2) is a minor isoform in the adult heart and is primarily associated with the embryonic heart. TPM2 has at least 2 splice variants: a skeletal muscle form (TB1-3) that includes exon 6B (also referred to as exon 7) and exon 9A, and a smooth muscle isoform (TM-1) that includes exon 6 (also referred to as 6A) and 9D. In the postnatal heart, β-tropomyosin expression is known to decrease, which was seen expectedly in the data. Moreover, we observed that this decrease primarily affected the skeletal muscle isoform TB1-3 (Figure 6F). This observation is corroborated by existing adult heart data showing the smooth muscle isoform TM-1 is predominantly expressed over the skeletal muscle isoform (67). A mutually exclusive exon pair 6 and 7 (also termed 6A and 6B) is known to be regulated by PTB, which suppresses exon 7 usage (68, 69). While this may appear incongruent with the postnatal decrease in PTBP1 in the heart, a recent meta-analysis of splice factor–knockout experiments revealed that TPM2 splicing requires the combined action of multiple splice factors, including RBM20, which suppresses exon 7/6B (67). Indeed, we observed a postnatal increase in RBM20 (limma FDR-adjusted *P* = 0.014) that may contribute to the decrease in the skeletal muscle TPM2-specific exons (Figure 6E).

PDLIM5 has several splice variants that have been referred to as the “long” and “short” isoform groups in the literature. Postnatal hearts shift toward PDLIM5-3 (Figure 6F), 1 of 2 shorter mouse PDLIM5 sequences documented on Swiss-Prot that omits residues 338–591 from the canonical PDLIM5 sequence. Therefore, it corresponds to a “short” PDLIM5 isoform and may correspond to the variant that is known to participate in cardiomyocyte binucleation during cardiac development (70). Paradoxically, whereas prior work has found that RBPMS deletion leads to the accumulation of the short isoform, we found that the postnatal increase in the short PDLIM5-3 spliceoform was associated with a slight but significant increase in RBPMS levels in postnatal hearts (Figure 6E), suggesting other factors may also influence PDLIM5 splicing.

Other changes include the alternative splicing of integrin β1, causing a relative expansion of the striated muscle 1D isoform (ITGB1-2) (71) over the canonical 1A (ITGB1) isoform (Figure 6F). Mitofilin (IMMT), which forms part of the MICOS complex in the mitochondrial inner membrane, shows a depletion of the uncharacterized IMMT-3 isoform in postnatal hearts (Figure 6F). Its splicing may also be influenced by RBM20 activity (72). In parallel, we observed a shift from SERCA2b (ubiquitous isoform) toward SERCA2a (muscle specific) (Figure 6F). While the SERCA2a isoform is known to be specific to slow-twitch skeletal and cardiac muscle, the results here provide further evidence that its differential splicing may form a part of the fetal gene program.

Finally, using the JCAST/MSFragger workflow (workflow 2), we further identify an isoform shift in QKI (Quaking, KH domain–containing RNA binding) (Figure 3G); QKI is a cardiac RBP that regulates alternative splicing and participates in wide-ranging processes from cardiomyocyte differentiation to ischemic injury. Meta-analysis of sequencing data showed that QKI regulates the splicing of important cardiac genes, including calmodulin-dependent protein kinase (CAMK2D), β-tropomyosin (TPM2), and titin (TTN) (67). QKI itself has 3 major splice isoforms: the designated canonical QKI-5, and the alternative isoforms QKI-6 and QKI-7 with different C-terminal residues. We found an increase in the QKI-6/QKI-5 ratio in the postnatal heart, which mirrors the decrease in QKI-5 that is seen over the course of embryogenesis in the brain (73). Whereas QKI-5 has a nuclear localization signal and is thought to regulate pre-mRNA splicing, QKI-6 is largely cytoplasmic (73) and may function in RNA stability or posttranscriptional regulations. In the heart, deletion of the *QKI* locus leads to abrogated cardiomyocyte contractility in human embryonic stem cells that is rescued QKI-5 but not QKI-6, supporting their functional diversification in cardiac cells (74). A change in QKI-5 and QKI-6 ratios from the same gene suggest QKI may participate in the rerouting of splicing networks during cardiac development alongside other RBP changes (16, 70). Taken together, notwithstanding continued challenges in spliceoform protein analysis, our results identified known and candidate alternative splicing isoform switches, demonstrating they provide a rich source of fetal gene candidates that merits further investigation.

### Fetal isoform ratios are partially reversed in pathological cardiac remodeling

**Figure 7:**
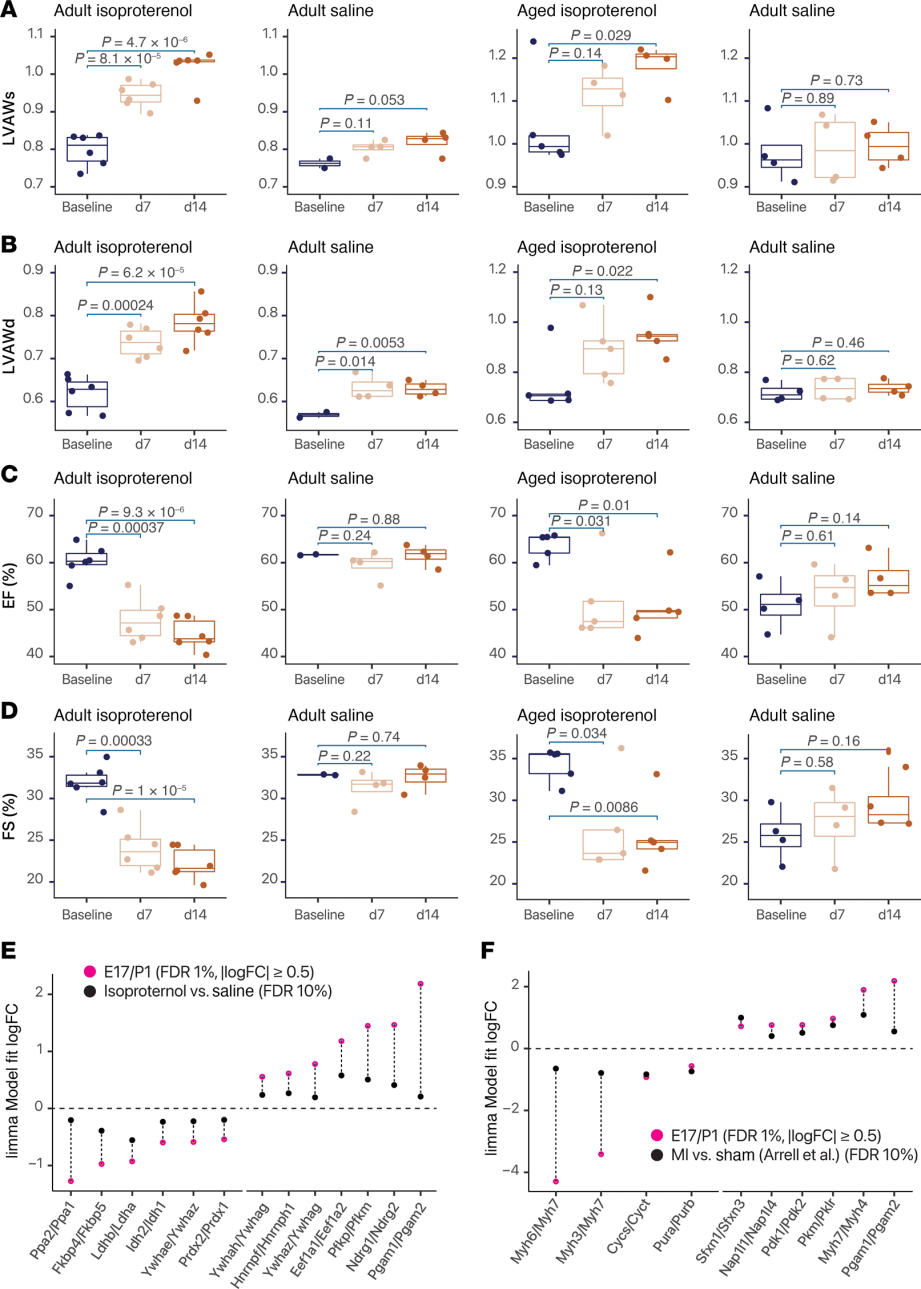
Partial return toward fetal isoform ratios in hypertrophic hearts. (**A**) Echocardiographic measurements of left ventricular systolic anterior wall thickness (LVAWs) at baseline, day 7, and day 14 of isoproterenol or saline administration for young adult (left) and aged (right) C57BL/6 mice. *P* values obtained from 2-sample *t* tests are indicated above bars between respective comparisons of day 7 versus baseline and day 14 versus baseline. (**B**) As in **A**, but for left ventricular anterior diastolic wall (LVAWd) thickness. (**C**) As in **A**, but for ejection fraction (EF%). (**D**) As in **A**, but for fractional shortening (FS%). (**E**) Protein log(fold change) (logFC) in fetal versus perinatal hearts (red) and adult hypertrophy versus baseline hearts (black). Paralog pairs that are differentially expressed in fetal hearts (FDR 1%, absolute logFC ≥ 0.5) and then showing codirectional changes in hypertrophy (FDR 10%) are included. For hypertrophic hearts, *n* = 4–7 per age/treatment group. (**F**) Protein logFC in fetal versus perinatal hearts (red) and mouse hearts 4 weeks after myocardial infarction (MI) versus sham surgery in Arrell et al. (77) (black). Paralog pairs that are differentially expressed in fetal hearts (FDR 1%, absolute logFC ≥ 0.5) and then showing significant codirectional changes in MI (FDR 10%) are included. For MI hearts, *n* = 4 per group.

To examine whether the isoform ratio shifts reverse in pathological cardiac remodeling, we generated an isoproterenol-induced model of pathological cardiac hypertrophy (Figure 7, A–D). On an individual-protein level, isoproterenol elevated the abundance of striated muscle contraction proteins and decreased mitochondrial metabolism and respiratory chain proteins, showing the experiments accurately captured the proteomic landscape of pathological hypertrophy (Supplemental Figure 5 and Supplemental Table 8). We then focused on which isoform pairs are significantly different (limma FDR-adjusted *P* ≤ 0.1) in hypertrophic hearts that show the same direction of change in fetal over postnatal comparisons, regardless of their log(fold change) (Supplemental Table 9). Under the applied threshold, we found several glycolytic proteins that show significant changes in isoform usage in hypertrophy and partially revert the direction of the fetal gene profiles, including a return to a lower LDHB/LDHA ratio (Figure 7E). Overexpression of LDHA in neonatal rat ventricular myocytes has been shown to drive hypertrophic cardiac growth in rodents (75), suggesting this particular isoform shift may contribute maladaptively to hypertrophy. In parallel, we observed a return toward a lower PFKP/PFKM ratio, the latter of which is known to be upregulated in failing human hearts and may be maladaptive (76). Other changes included a return toward a higher PGAM1/PGAM2 ratio; a return toward a lower PPA2/PPA1 ratio, important metabolic proteins in the mitochondria that have not been explored in the context of cardiac development or remodeling; and a return toward higher EEF1A1/EEF1A2 ratios, 2 homologous members of the eEF1A component of the eEF1 complex with unclear distinction of function. In each case, however, only a partial reversal was observed (Figure 7E).

To corroborate this partial reversal, we applied the protein ratio calculations to a label-free mass spectrometry dataset by Arrell et al. (77) who performed left anterior descending coronary artery ligation to induce myocardial infarction in mice and then measured cardiac remodeling after 4 weeks. We likewise observed only a partial reversion to the fetal program in PGAM1/2 and PDK1/2 that was lower in amplitude than the fetal/perinatal changes (Figure 7F). Thus, the incompleteness of fetal gene reactivation at the protein level cannot be fully explained by the isoproterenol model or the ratio compression effect of TMT labeling.

### Isoform ratio quantification in human cardiomyocyte differentiation

**Figure 8:**
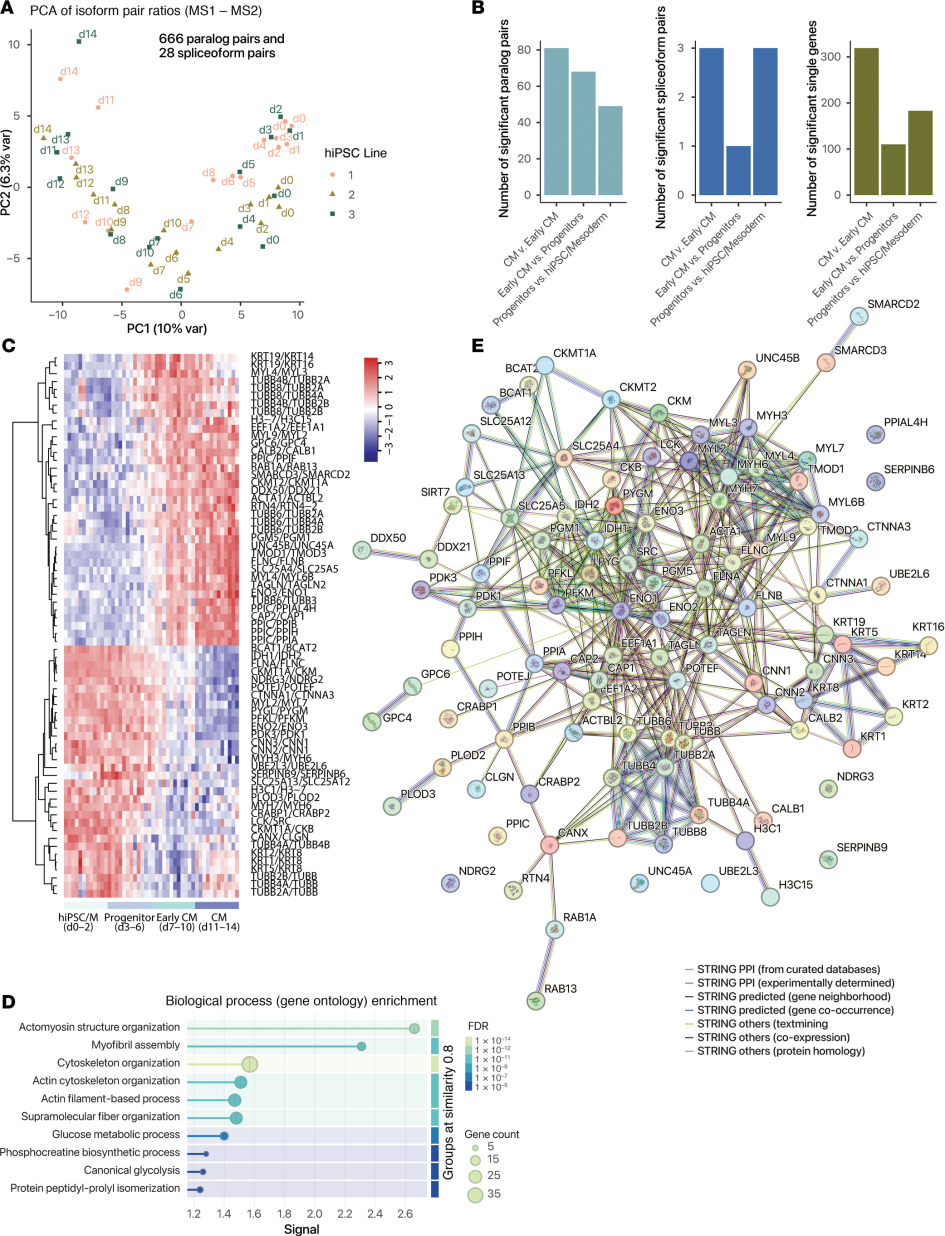
Proteome-wide protein isoform ratio quantification in hiPSC cardiomyocyte (CM) differentiation. (**A**) PCA of the ratios of 666 paralog pairs and 28 spliceoform pairs largely distinguished hiPSC differentiation stages. Colors: biological replicate lines. (**B**) Number of statistically significant (limma FDR-adjusted *P* < 0.01, MIF ≥ 0.05) paralog pairs (left); spliceoform pairs (middle); and individual proteins (right) at each stage of differentiation, from hiPSC/mesoderm (days 0–2) to progenitors (days 3–6), early-CM (days 7–10), and CM (days 10–14). (**C**) Heatmap showing isoform pairs with significantly different usage in early-CM to CM transition. Colors: row-standardized ratios. (**D**) STRING enrichment (see Supplemental Methods) graph of proteins with differential isoform usage. Colors: FDR. (**E**) STRING network graph of proteins with differential isoform usage. Edge colors: STRING interaction type.

Finally, we asked whether proteome-wide ratio tests can be applied to other datasets as a generalizable approach to gain insights into gene regulation programs. To do so, we reanalyzed a set of TMT-labeled mass spectrometry data on the proteomes of human induced pluripotent stem cells (hiPSCs) during cardiomyocyte differentiation (59). Briefly, 3 hiPSC donor lines were directed to mesoderm specification, cardiac progenitors, and cardiomyocytes using a small molecule–based protocol (59). Cellular proteomes were analyzed daily from day 0–14 of differentiation (Supplemental Table 10). Using the isoform ratio calculation workflow, we quantified the log ratios of 666 pairs of paralogs and 28 pairs of spliceoform proteins (Supplemental Table 11). Isoform ratios readily distinguish cells by differentiation days and stages (Figure 8A). We then used the linear model and empirical Bayes methods in limma to compare isoform shifts across 4 differentiation milestones: (a) hiPSC to mesoderm (days 0–2 of differentiation), (b) mesoderm to cardiac progenitor (days 3–6), (c) early-cardiomyocyte (days 7–10), and (d) maturating cardiomyocyte (days 11–14) (Supplemental Figures 6 and 7, and Supplemental Tables 12–14).

Our analysis showed that when contrasted with the changes in the other differentiation stages, the transition from progenitor cells to early-cardiomyocytes preferentially alters isoform usage over single-protein changes. Thus, early-cardiomyocyte specification appears to be a critical point of substantial isoform usage rewiring (Figure 8B). Inspection of the significant protein pairs at this stage revealed a number of isoform shifts that mirror mouse heart development, including differing usages in ENO2/ENO3 and MYH7/MYH6 (Figure 8C). On a pathway level, differential isoform usages in early-cardiomyocytes are enriched in actomyosin structure organization, myofibril assembly, and glucose metabolic process (Figure 8D). Likewise, STRING network analysis showed distinct clusters of metabolic (e.g., PGM1, IDH1, PFKM), myofibril (MYL2, MYH6, MYH7), and cytoskeletal (e.g., tubulins, keratins, and calponins) proteins with isoform usage changes (Figure 8E). Conversely, the mesoderm to progenitor stage features isoform usage changes in proteins associated with phosphocreatine process and Wnt signaling pathway but not cytoskeletal terms (Supplemental Figure 8). Lastly, early-cardiomyocyte to cardiomyocyte transition shows prominent involvements of proteins in myofibril assembly and striated muscle development, but not metabolic annotations (Supplemental Figure 9). We surmise that hiPSC-cardiomyocyte differentiation is associated with waves of isoform usage changes, each associated with different biological pathways and processes. Moreover, the analysis demonstrates the potential of proteome ratio tests to provide insights in new datasets and biological systems.

## Discussion

This work presents the first proteomics study to our knowledge to systematically examine the ratiometric changes in protein isoform pairs in the fetal heart. The ratios between protein isoforms can reveal reciprocally regulated relationships between 2 closely related homologous proteins. Paralog genes and alternative splicing present 2 major modalities from which new protein function is created. Paralogs arise from duplication events of ancestral genes or genomes, which allow the ancestral copy to retain its function while leaving the new copy free to be selected for new expression and function across tissues and developmental stages (40). A deep parallelism has been noted between the acquisition of new functions by differential gene regulation and differential alternative splicing. Both mechanisms have harnessed duplication events (gene duplication and exon duplication) during bilaterian evolution to allow extra copies of genetic material to evolve specialized function, and both can occur through new interactions between genetic elements (promoters and splice sites) and protein regulators (transcription factors and splice factors) in a context-specific manner (78). A protein (group) can also be regulated through the expression of both paralogs and alternative splicing isoforms (e.g., tropomyosins and troponins).

Applying the protein ratio tests to the fetal heart, we found numerous well-established cardiac fetal gene pairs in the data. In terms of magnitude of protein proportion changes, hallmark changes in contractile proteins presented the most prominent usage shifts in the fetal heart (i.e., with the greatest log[fold changes]), followed by glucose metabolism proteins. This may explain the prior emphasis on these pathways. Nevertheless, smaller-magnitude changes can also effectuate biologically important outcomes, and the ratios between 2 reciprocally regulated or mutually regulating proteins are sensitive to even small differences in protein pool proportions –– a 60:40 to 40:60 proportion shift leads to greater than 2-fold changes in 2 proteins’ relative abundance. Indeed, we uncovered multiple protein isoform pairs that to our knowledge were not considered part of the fetal gene program and may reveal new insights into cardiac development and disease. For instance, our data implicate multiple mitochondrial protein pairs in cardiac development, suggesting the shift away from carbohydrate metabolism as the preferred fuel source is paralleled by a concurrent remodeling of mitochondrial protein compositions. These changes involve paralogs like inorganic pyrophosphatases PPA1/PPA2, ADP/ATP translocases ANT1/ANT2 (SLC25A4/SLC25A5), and VDAC1/VDAC2; as well as alternative isoform pairs like GLS1 variants (KGA/GAC).

Changes in isoform usage could affect function by modulating protein enzymatic activity and interaction partners, even in cases where the isoforms share highly similar sequences and domain structures. For instance, while the β and α myosin heavy chains (MYH7 and MYH6) share a remarkable degree of sequence homology (79) (~93% identity in the mouse), the fast α isoform has up to 3-fold higher ATPase activity (80); the shift from MYH7 to MYH6 is consistent with postnatal adaptations toward higher contractile activity. For enolases ENO1 and ENO3 (~84% sequence identity), enzymes purified from the liver (presumed ENO1 or α-/liver enolase) and the muscle (presumed ENO3 or β-/muscle enolase) show similar *K*_m_ with 2-phospho-D-glycerate in the forward glycolysis direction at physiological pH, yet the muscle isozyme has stronger affinity (lower *K*_m_) for phosphoenolpyruvate (81), which may support versatile metabolic controls in postnatal hearts. In the case of PCBP1 and PCBP2 (~85% sequence identity), a domain-by-domain view suggests that the 3 RNA-binding KH domains KH1 (residues 13–75 in PCBP1), KH2 (residues 97–162), and KH3 (residues 279–343) are highly conserved, whereas the linker between KH2 and KH3 is more variable. This suggests the core RNA-binding machinery of the proteins is unchanged but the linker differences could affect flexibility between domains and the ability to form interactions. Indeed, our overexpression of PCBP1 versus PCBP2 in vitro led to correlated but nonidentical transcriptome profiles (*r* = 0.59, *P* ≈ 0), suggesting they affect similar but incompletely overlapping sets of genes.

Among mitochondrial proteins, the preference of ANT1 over ANT2 in postnatal hearts suggests adaptations of ATP export in a highly metabolically active state, but may also have implications in cell death susceptibility given the role of ANT1 in cardiac cell apoptosis (82). Likewise, while the function of VDAC isoforms remains to be elucidated (83), there is evidence showing VDAC1 may promote apoptosis (84), while VDAC2 may inhibit apoptosis (85); hence, the dual increase in VDAC1/VDAC2 and ANT1/ANT2 ratios could reflect a postnatal change in mitochondrial physiology that alters cell death susceptibility. Further interpretation of the biological consequences of the isoform usage shifts would require functional studies. Such analysis may be aided by domain analysis to identify regions of sequence differences between isoforms, as well as consideration of isoform-specific enzyme activity or binding affinity, where such data are available.

In the hypertrophic mouse heart experiment, we found that only some fetal gene shifts are reversed and they do not revert fully to the protein pool compositions of E17 hearts when seen in the light of protein abundance proportions. Although the small sample size investigated here and differences between animal disease models can limit the number or magnitude of fetal gene reactivation that we identify, the results nevertheless suggest that the return to the fetal gene program in pathological hearts may be partial in amplitude at the protein level. This may reflect buffering of gene expression changes in posttranscriptional and posttranslational layers. In a second application, proteome-wide ratio tests in hiPSC-cardiomyocyte differentiation reveal a parallel shift in protein ratios in human cardiac differentiation and demonstrate the potential utility of the method in different species and models.

Finally, we consider some practical advantages of explicitly testing for ratio differences rather than individual protein levels in proteomics experiments. In recent work, Suhre suggested that in a genome association study, accounting for ratio associations with genetic variants can lead to significant increases in power (“p-gains”) by revealing the shared genetic and nongenetic variances between 2 genes, e.g., by uncovering a trans-QTL that simultaneously induces one gene while suppressing another (35). In the case of quantitative proteomics, we posit that such a contradirectional modulator would have the effect of amplifying ratio changes over the effect size of individual protein fold changes and by doing so increase the power of discovery. Moreover, in a mass spectrometry experiment, both latent biological (e.g., unaccounted for age or sex differences) and technical (e.g., chromatographic variations and batch effects) factors may exert a similar effect over 2 proteins, which may then be effectively corrected by comparing their ratios between conditions. This self-normalization effect likely also motivated classic biochemical studies to consider the ratios of 2 proteins in targeted studies.

### Limitations of the study.

As we focused on comparing the ratios between 2 isoforms, this study omitted isoforms that behave similarly to each other in fetal and adult hearts. Fetal genes that are not part of a paralog or spliceoform group (e.g., ANP) were likewise excluded.

Secondly, MS2 TMT quantification can lead to ratio compression due to co-eluting isobaric peptides, which may bias measured ratios toward 1:1. This may be alleviated with MS3-based TMT quantification, although prior works found only minimal benefits on the accuracy and interpretation of hyperplexed experiments (86, 87). The ratio comparison concept is readily applied to label-free quantification data. Other general limitations of proteomics include the still-incomplete coverage of all proteins, sample size constraints, and differences between bulk versus single-cell proteomes. These issues can be addressed with continued advances in technology.

Thirdly, in the ratio tests, a protein measurement may appear in multiple ratios (i.e., A/B and A/C), which introduces nonindependence to the data matrix. To partially account for this dependency, we used a more stringent 1% limma FDR-adjusted *P* value, compared with the common 5% threshold. The effect of additional filtering and normalization steps was not investigated here.

Finally, additional experiments are needed to validate the fetal gene candidates. Isoform usage may also differ between humans and mice, e.g., α to β switch in human ventricles. While we showed using hiPSC data that the ratio tests can be applied to human cells, the translatability of findings to human heart development remains to be elucidated. These limitations suggest valuable areas of future development.

### Conclusion.

In summary, we describe a ratiometric proteomics workflow to discover differences in protein isoform usage across samples. Applying this workflow to the mouse heart, we identified hallmark fetal genes and potential new candidates, providing a resource of the fetal gene program of the heart at the protein level. More generally, this work highlights the prospects of applying protein ratio tests as a data analytical strategy of proteomics data, which should find utility in the discovery of gene regulatory mechanisms beyond individual-protein changes.

## Methods

Further information can be found in Supplemental Methods.

### Sex as a biological variable.

For commercially procured fetal and perinatal hearts, specimens were not genotyped; it is unknown whether similar findings would be observed in male and female hearts. For the hypertrophy study, our study examined male mice to reduce potential variability in phenotype. It is unknown whether the findings are relevant for female mice.

### Statistics.

To perform statistical tests of either single protein or isoform ratios, we used the empirical Bayes and moderated *t* test implemented in the limma package v.3.58.1 (88) on the protein abundance data frame or the log difference of protein isoform data frame, respectively. The design matrix and linear model used the formula, expression ~ age (E17 vs. P1) for the fetal/postnatal comparison; for hypertrophy, expression ~ treatment (isoproterenol vs. sham) + age (young adult vs. old) + TMT block was used. Diagnostic plots for data linearity and distributions were performed using the built-in limma plotMA(), plotSA(), and plotMD() functions. Unless otherwise stated, a limma FDR-adjusted *P*-value cutoff of 0.01 (1% FDR) was considered significant for fetal comparisons, along with an absolute log(fold change) of 0.5 or greater. A limma FDR-adjusted *P* of 0.1 (10% FDR) was considered significant for hypertrophy comparisons.

### Study approval.

All animal protocols were approved by the Institutional Animal Care and Use Committee (IACUC) guidelines at the University of Colorado Denver/Anschutz Medical Campus.

### Data availability.

Mass spectrometry data and the associated metadata have been deposited to ProteomeXchange at PXD052640 and PXD052641. hiPSC cardiomyocyte differentiation data are on ProteomeXchange at PXD013426. RNA sequencing data have been uploaded to figshare under doi:10.6084/m9.figshare.28830053, and deposited to the NCBI GEO with the accession GSE303619. Values used for generation of graphs in figures are included in the Supporting Data Values file.

## Author contributions

YH, SB, SAW, and MPYL designed the original investigations and acquired the data. EL and MPYL analyzed the data and drafted the manuscript. BP and DCMN contributed additional research designs. All authors contributed to the submitted version of the manuscript. EL and MPYL handled funding.

## Supplementary Material

Supplemental data

Supplemental table 1

Supplemental table 10

Supplemental table 11

Supplemental table 12

Supplemental table 13

Supplemental table 14

Supplemental table 2

Supplemental table 3

Supplemental table 4

Supplemental table 5

Supplemental table 6

Supplemental table 7

Supplemental table 8

Supplemental table 9

Supporting data values

## Version 1 08/07/2025

In-Press Preview

## Version 2 09/23/2025

Electronic publication
