# Supplementary material for: A ratiometric catalog of protein isoform shifts in the cardiac fetal gene program
Source: JCI Insight. 2025 Aug 7;10(18):e184309. doi: 10.1172/jci.insight.184309 (PMC12487847; doi:10.1172/jci.insight.184309)
Supplement: Supplemental data [file jciinsight-10-184309-s163.pdf]

**Supplemental Material**

# A Ratiometric Catalog of Protein Isoform Shifts in the Cardiac Fetal Gene Program

[1,2]*Yu Han, [1,2]*Shaonil Binti, [1,2]Sara A. Wennersten, [1,2]Boomathi Pandi, [1,2]Dominic C. M. Ng, [1,2]Edward Lau, [1,2,3]Maggie P. Y. Lam

1. Department of Medicine
2. Consortium for Fibrosis Research and Translation
3. Department of Biochemistry and Molecular Genetics

University of Colorado School of Medicine
Aurora, CO 80045, USA

* Equal contributions

**Supplemental Text S1**

Rationale

Although MS2 multiplexing increases scalability and reduces variance in quantitative comparisons, TMT reporter channel intensities represent relative abundance of the same peptide across different samples, and therefore do not directly lend to comparisons between distinct proteins (1). To compare the absolute quantity of two proteins and thereby calculate their ratios across conditions, we therefore implemented a workflow that combines the MS1 precursor ion intensity of each peptide and the proportional abundance of their MS2 TMT reporter channels. Briefly, the MS1-level precursor chromatographic intensity contains across-peptide intensities, whereas the MS2-level TMT reporter intensity of each peptide contains across-sample intensities across experiments. The basic intuition is that the precursor ion quantity of a peptide at the MS1 level can be distributed to multiple samples based on their proportional MS2 reporter intensity (2–5). The resulting composite MS1-MS2 intensity would then allow us to leverage MS2-based multiplexing while still acquiring the absolute protein quantities needed to calculate protein ratios. To do so, we first performed database search of TMT-labeled mass spectrometry data, then extracted MS1 and MS2 intensities for each protein. We then extracted paralogs pairs and splice isoform pairs of each quantified protein and used their log ratios for statistical tests (see Supplemental Methods).

An additional potential advantage of ratio quantification is that by measuring the absolute abundance of isoforms within an isoform group, we reveal information about proportional pool sizes that can be useful for data interpretation and prioritization. This contrasts with using the fold-change (i.e., up/down regulation) of individual proteins alone, which can obscure drastic differences in the copy numbers of two closely related proteins, in a manner analogous to the stoichiometric occupancy of post-translational modification sites vs. fold-change of modified peptides. In other words, a minor isoform may be expressed in such a miniscule level, that even up-regulated, may be of insufficient quantity to exert a functional outcome (e.g., a minor splice isoform that exists at 0.1% of the the canonical isoform will still only account for 0.4% of the protein pool after 4-fold up-regulation).

**Supplemental Methods**

**Animal Experiments**

Fetal and postnatal C57BL/6J mouse hearts (n = 5 each) were acquired commercially at E17 and P1 from Zyagen (San Diego, CA), weighed, and homogenized for proteomics profiling (see below). For normal and hypertrophic adult mouse hearts, wild-type C57BL/6J mice were purchased from Jackson Laboratories (Bar Harbor, ME, USA) and housed in a temperature-controlled environment on a 12-h light/dark cycle and with free access to normal chow diet and water under National Institutes of Health (NIH) guidelines for the Care and Use of Laboratory Animals. Male adult (15–16 wk) and aged (83–84 wk) mice were administered with saline or 20 mg/kg isoproterenol by micro-osmotic pump (ALZET) implantation for a duration of 7 or 14 days as previously described (6) (n = 4, 7, 4, 6 for young adult saline 7 days, young adult isoproterenol 7 days, aged saline 7 days, aged isoproterenol 7 days; n = 4, 6, 4, 5 young adult saline 14 days, young adult isoproterenol 14 days, aged saline 14 days, aged isoproterenol 14 days, 40 male animals total). The animals were euthanized, followed by measurement of body weight, heart weight and tibia length. Left ventricles were collected and kept frozen at –80 °C.

<u>Echocardiography</u>

Transthoracic echocardiograms were performed using the VisualSonics Vevo2100 system (Visualsonics, Toronto, Canada). Animals were anesthetized in an induction chamber at 2% isoflurane. Once appropriately sedated, mice were transferred to the imaging platform. Hair on the chest was removed with a depilatory lotion and the mouse was secured by taping all four limbs to the platform. Body temperature was maintained at 37°C and isoflurane was delivered through a nose cone at 1.5% throughout the session. Mice were under anesthesia for no longer than 15 minutes. SAX views of the LV at the papillary muscle level were used to acquire M-mode images. Left ventricular anterior wall (LVAW) thickness in systole and diastole was measured using the M-mode images in order to measure changes in wall thickness and systolic function. Heart rate was measured in each image and averaged. All measurements were averaged from at least three cardiac cycles during the exhale phase and taken at body temperatures between 37°C and 38°C. All echocardiographic measurements and analyses were performed in a blinded manner.

**Protein Chemistry and Mass Spectrometry**

Tissue homogenization was performed with a handheld homogenizer (OMNI International). Samples were placed on ice for 1 minute to allow cooling down. Repeat for a total of 3 rounds of homogenization. Homogenized tissues were sonicated with a handheld sonicator (Fisher Model 120), at 40% amplitude for 1 s, pause for 5 s, for 15 cycles. The samples were then centrifuged at 5,000 × g for 1 min at 4°C, and vortex for 10 s, and repeated for three rounds of sonication and centrifugation. The extracted protein samples were further centrifuged at 14,000 × g for 15 min at 4°C to collect supernatants and measure protein concentration with the BCA protein assay kit following manufacturer instructions. The samples were then reduced, alkylated, and digested with sequencing-grade trypsin as previously described (7). The digested peptides were labeled with TMT 10-plex isobaric tags (Thermo), and fractionated using high-pH reversed-phase columns (Pierce) following manufacturer instructions.

Mass spectrometry was performed on a Q-Exactive HF orbitrap high-resolution mass spectrometer (Thermo) coupled to a Thermo Easy-nLC UPLC liquid chromatography system. For fetal heart samples, The LC gradient was as follows: 0 – 105 minutes, 0 – 40%B; 105 – 110 minutes, 40– 70% B, 110 – 115 minutes: 70 – 100% B; 115 – 120 minutes: hold; at 300 nL/min. Mass spectrometry data were acquired in data dependent acquisition (DDA) mode using typical settings including 60,000 MS1 and MS2 resolution, 3e6 MS1 AGC; 2e5 MS2 AGC; 100ms maximum IT; 15 TopN; isolation window 1.4 m/z; normalized CE 28, 30, 32; and 30 seconds dynamic exclusion. For cardiac remodeling samples, The LC gradient was as follows: 0 – 105 minutes, 0 – 30% B; 105 – 110 minutes, 30 – 70% B, 110 – 115 minutes: 70 – 100% B; 115 – 120 minutes: hold; at 300 nL/min. Mass spectrometry data were acquired in data dependent

acquisition (DDA) mode using typical settings including 60,000 MS1 and MS2 resolution, 3e6 MS1 AGC; 2e5 MS2 AGC; 110 ms maximum IT; 15 TopN; isolation window 0.7 m/z; normalized CE 28, 30, 32; and 30 seconds dynamic exclusion.

**Data Analysis**

<u>Mass spectrometry database search</u>

Mass spectrometry data was searched using Sage v.0.14.5 (8) aarch64-apple-darwin build to identify proteins and to measure peptide MS2 TMT and MS1 LFQ (label-free quantification) intensity, using typical settings including: missed_cleavages: 2, precursor_tol: –20 to +20 ppm; fragment_tol: –20 to +20 ppm; isotope: false; static_mods: C 57.0215, K 229.1629; variable mods: M 15.9949, Peptide N-terminus 229.1629, S 229.1629; generate_decoys: true; isotope_errors: 0 – 3; quant: tmt: Tmt10; quant: lfq: true; predict_rt: true. For mouse fetal and hypertrophy heart samples, the database used was retrieved on 2023-09-15 from UniProt (9) to contain 25,530 Swiss-Prot *Mus musculus* reviewed canonical and isoform entries. A peptide-level multiple testing adjusted q value cutoff of 0.01 was used for confident identification. For hiPSC data re-analysis, raw mass spectrometry files were retrieved from PXD013426. The database used was UniProt Swiss-Prot *Homo sapiens* canonical and isoform entries appended with JCAST as described in the original study (10). Sage settings include static_mods: C 57.0215, K 229.1629; variable mods: M 15.9949, Peptide N-terminus 229.1629; chimera: true; report_psms: 2. Other search settings were as described above. A peptide-level multiple testing adjusted q value cutoff of 0.01 was used for confident identification.

<u>Calculation of MS1 and MS2 intensities</u>

Peptides passing the q value cutoff and matching to non-decoy entries are used for quantification. For each peptide, their TMT reporter intensity is corrected for isotope spillover using non-negative least squares (11) as described (12), with the isotope purity matrix matching the TMT batch from the manufacturer. To overcome the issue of sequence redundancy, i.e., most peptides are not unique to a single database entry due to the high degree of similarities between canonical and alternative isoforms, we applied a protein isoform rollup routine for parsimony. The rationale of this workflow follows precedents in the protein inference literature (13) and has also been employed in a prior study from our group (14). Briefly, it assumes that most alternative isoforms are not expressed or only present in very low concentration unless evidence suggests otherwise. Peptides that are shared across two proteins coded from the same gene are therefore assigned to the canonical form only, unless the alternative isoform possesses one or more uniquely mappable peptides in the database search result. All peptides that are uniquely mappable to a single UniProt isoform entry, or uniquely mappable to a single UniProt canonical entry and where the non-canonical entries have no unique peptides, are used for quantification. The TMT channel intensities of all constituent peptides for each protein for each sample are then summed.

We next retrieve the MS1 intensities of each peptide from the Sage “lfq.tsv” output, which are then filtered and summed for each protein as above. We then perform a first-pass protein MS1 quantity calculation. To account for a longer protein emitting give more label free intensity than an equimolar shorter protein, we divide the sum of MS1 intensities of all peptides that are uniquely mappable to a protein by the total protein sequence length retrieved from the FASTA database used in the search, which gives a near-identical normalization across log-log scale as division by number of theoretically observable tryptic peptides (data not shown).

Next, we calculate a second-pass distributed MS1 quantification value, by considering the intensities of all non-uniquely mapped peptides. Briefly, the MS1 intensities of peptides shared by multiple proteins are divided proportionally by the unique-peptide abundance as calculated n the first-

pass (unique-peptide-only) MS1 quantification value of each of the shared protein, then added to the MS1 quantification value of each respective protein. The total MS1 intensity values of each sample are then normalized by column sum, and missing values are median-imputed. Finally, we distribute the MS1 quantification value of each protein across samples using the TMT matrix calculated above. The TMT matrix is row-wise normalized to 1 to compute a sample proportional abundance matrix, then multiplied to the second-pass MS1 quantification value to yield the final MS1-MS2 protein abundance. Rows (proteins) with only missing values (NA) are removed. For downstream statistical testing, the protein abundance values are normalized by variance stabilizing transformation to stabilize the mean-variance relationship in the data, by using the limma::normalizeVSN() function for the fetal data (which has only 1 TMT block). For the hypertrophy data, which has 5 TMT blocks and contains shared reference channels, an internal reference normalization using two reference channels is further carried out prior to variance stabilizing transformation. The normalized data are inspected using boxplots and PCA plots for all samples.

Comparison of protein abundance estimates

For comparison to other estimates of protein abundance, we convert the absolute quantification values to copy number per cells, based on $10^9$ total protein molecules per cell, and compared to to the iBAQ absolute quantification value- based copy number in NIH 3T3 mouse fibroblasts via identical gene names as reported in the Supplemental Table S3 of (15). To further validate the MS1-MS2 based (i.e., TMT/LFQ) protein quantity values, we convert the composite protein quantities to proportional copy numbers/absolute abundances in p.p.m., and compared these values to absolute protein copy numbers in the mouse heart compiled in PaxDB (16), which is a protein quantity database compiled from the aggregation of existing data sets including mass spectrometry data from multiple studies in mouse heart tissues (17–19).

Paralog and splice isoform identification and ratio calculation

Paralogs are retrieved from the Ensembl (20) *Mus musculu*s data set using biomaRt (21) with the getBM() function, retrieving the “ensembl_gene_id”, “external_gene_name”, “mmusculus_paralog_ensembl_gene”, “mmusculus_paralog_associated_gene_name”, “mmusculus_paralog_canonical_transcript_protein”, “mmusculus_paralog_orthology_type”, “mmusculus_paralog_subtype”, “mmusculus_paralog_perc_id” and “mmusculus_paralog_perc_id_r1” attributes. Using the “mmusculus_paralog_perc_id” and “mmusculus_paralog_perc_id_r1” fields, we then filter in only paralogs with 50% or higher bidirectional gene sequence identity between two paralogs within a pair. Paralog pairs that map to identical UniProt accessions or contain missing accessions are removed. Orthology type is retrieved from the “mmusculus_paralog_orthology_type” and “mmusculus_paralog_subtype” fields. We then identify all disjoint paralog groups within the paralog tables using gene names that are explicitly identified in the fetal proteomics experiment. Within each group, we then calculate the group size, average sequence identity, and the ratios of MS1-MS2 protein abundance within each sample. For statistical testing, from the variance stabilization transformed data, the difference in normalized expression ($x_1 - x_2$) between two isoforms within a group were calculated to represent the generalized log ratio of the two proteins. The data table containing only either single-protein values or isoform ratios were then used for comparisons. See main text for statistical tests.

The ratios of splice isoforms within isoform groups are calculated analogously, by identifying UniProt isoform accession (e.g., P#####-2, P#####-3, etc.) belonging to the same canonical UniProt accession (P#####). To corroborate the protein quantification values and to further identify splicing-derived isoforms, we further performed a database search using a computational strategy we previously developed to enable RNA-seq-guided proteomics to identify protein isoforms from the mass spectrometry data (10, 22). Briefly, a custom database was generated using JCAST v.0.3.4 (23) with deep RNA sequencing data generated from the mouse heart as described (7). The non-canonical sequences were

then appended to the Swiss-Prot canonical + isoform database to yield a total of 29,102 forward entries. This database was used to repeat the database search using MSFragger v.3.8 (24) using typical settings, including precursor_mass_lower: –20 ppm; precursor_mass_upper: 20 ppm; fragment_mass_tolerance 20 ppm; isotope error: 0/1/2; allowed_missed_cleavage: 2; num_enzyme_termini: 1. The search results then underwent confidence determination using MSBooster v.1.1.6 (25) and Percolator v.3.0 (26). The MS2 and MS1 intensity of confidently identified peptides (Percolator q value < 0.01) were then extracted using pyTMT v.0.4.1 (12) and Riana v.0.7.1 (27), respectively. Protein abundance for spliceoform pairs was then calculated as above.

Additional data analysis

Functional enrichment analysis was performed using gene set enrichment analysis (GSEA) against Reactome annotated pathways (28) using the ReactomePA package (29). Enriched gene sets/annotations with FDR adjusted permutation test P value < 0.01 (1% FDR) are considered significant. Additional data analysis is performed with the aid of common R packages in the tidyverse and on Bioconductor, and the STRING-db (30) website functions.

## Immunoblots

Hearts from E17 and P1 stage C57BL/6 mice (n=3 each) were lysed in RIPA buffer (Cat# 89901, Thermo Scientific) supplemented with protease inhibitor (Cat# 78442, Thermo Scientific) and homogenized using a bead mill homogenizer (Omni Bead Ruptor 4) at speed 5 for 20 seconds. Homogenized tissues were then further sonicated using a handheld sonicator probe for 15 pulses (1s on/1s off, 40% amplitude). Crude lysates were centrifuged at 13, 500 g for 15 minutes and supernatant was collected into a clean tube. Digital immunoblot analysis of the protein lysates was performed in the Jess Simple Western System (ProteinSimple) using the 12–230 kDa Separation module (Cat# SM-W004, ProteinSimple), according to the manufacturer's instructions. The following primary antibodies and the dilution ratios were used in the Jess Simple Western: ENO1 (Cat# 11204-1-AP, Proteintech; 1:100), ENO3 (Cat# 55234-1-AP, Proteintech; 1:200), PGAM1 (Cat# NBP1-49532, Novus Biologicals; 1:40), PGAM2 (Cat# 15550-1-AP, Proteintech; 1:10), PPA-1 (Cat# 14985-1-AP, Proteintech; 1:10), PPA-2 (Cat# 16662-1-AP, Proteintech; 1:50), GAC-specific (Cat# 19958-1-AP, Proteintech; 1:50), and KGA-Specific antibody (Cat# 20170-1-AP, Proteintech; 1:50). Normalization was performed using a protein normalization reagent (Cat# DM-PN02, ProteinSimple) according to the manufacturer's guidelines. Data analysis was performed using the Compass software (version 6.1.0) for Simple Western. Unless otherwise stated, a two-sample, two-tailed t-test is used for statistical tests for immunoblots data.

## RNA sequencing

AC16 cells were seeded into 6-well plates in DMEM/F12 supplemented with 10% fetal bovine serum. Next day, when the cells reached ~70% confluency, they were transfected with PCBP1::Halo plasmid, PCBP2::Halo plasmid, or both at a concentration of 1 mg of each plasmid in 2 mL media using Lipofectamine 2000 (Cat# 11668019, Invitrogen) transfection reagent. Untransfected cells served as control. After 6 hours of incubation, the media containing the transfection reagent was replaced with fresh media. Cells were lysed with Trizol (Cat# 15596018, Invitrogen) 48 hours post-transfection. Lysates were centrifuged at 13500 × g for 5 minutes and RNA extraction was performed using Direct-zol RNA Kit (Cat# R2050, Zymo). RNA yield was measured using a Qubit fluorometer (Thermo) and RNA integrity score was measured using TapeStation (Agilent). PolyA enrichment, library construction, and short-read sequencing were carried out at Novogene USA. Sequencing was performed in paired-end mode with a read length of 150 base pairs and depth of ~20 G per sample on an Illumina NovaSeq platform. The RNA-seq data were then aligned against GRCh38.p14/GENCODE v47 (31) using STAR v.2.7.11a (32), with –sjdbOverhang 149 and typical settings. Transcript assembly was performed using StringTie v.2.1.1 (33), and differential expression analysis using DESeq2 (34) using typical settings. Results with apeglm

(35) shrinkage s-value < 0.05 and abs(logFC) > 0.25 are considered significant.

## Supplemental Tables

**Table S1** - Data of all quantified absolute protein abundance in the mouse E17 vs. P1 heart data set

**Table S2** - Data of all quantified protein isoform ratios in the mouse E17 vs. P1 heart data set

**Table S3** - Results of statistical tests of protein paralog ratios in the mouse E17 vs. P1 heart data set

**Table S4** - Results of statistical tests of individual protein expression in the mouse E17 vs. P1 heart data set

**Table S5** - Protein paralog pairs with significant shifts in isoform usage but not individual protein expression

**Table S6** - Evolutionary conservation and sequence identity of Ensembl retrieved paralogs from all quantified paralog groups

**Table S7** - Alternative splicing isoforms with significant difference in in the mouse E17 vs. P1 heart data set

**Table S8** - Results of statistical tests of individual protein expression in the mouse isoproterenol-induced hypertrophy data set

**Table S9** - Results of statistical tests of protein isoform ratios in the mouse isoproterenol-induced hypertrophy data set

**Table S10** - Data of all quantified absolute protein abundance in the hiPSC differentiation data set

**Table S11** - Data of all quantified protein isoform ratios in the hiPSC differentiation data set

**Table S12** - Results of statistical tests of protein isoform ratios in hiPSC/mesoderm vs. cardiac progenitor cells

**Table S13** - Results of statistical tests of protein isoform ratios in early hiPSC-cardiomyocytes vs. cardiac progenitor cells

**Table S14** - Results of statistical tests of protein isoform ratios in hiPSC-cardiomyocytes vs. early hiPSC-cardiomyocytes

**Supplemental Figures**

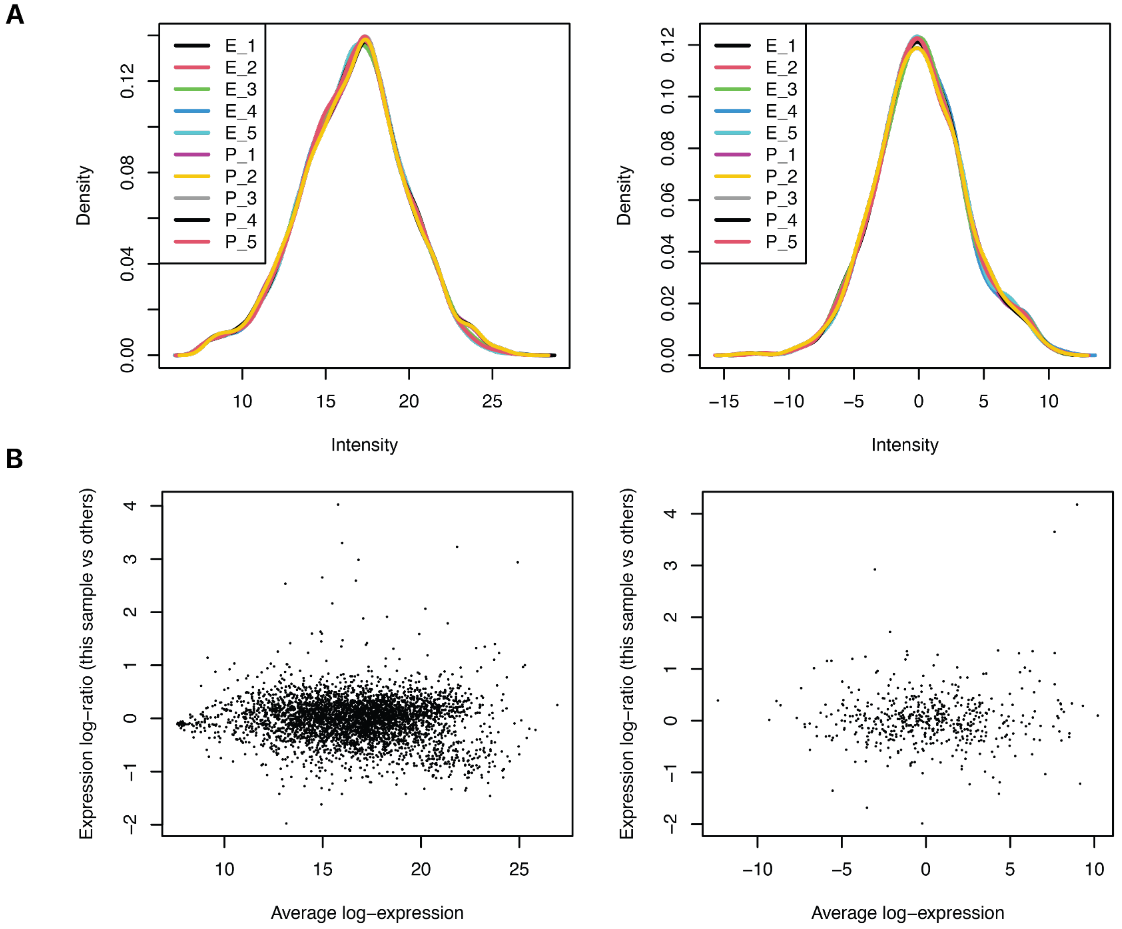


**Figure S1**. **Comparison of limma diagnostic plots in protein and ratio analysis.**
**A.** Distribution of generalized log intensity among all individual protein species (left) and protein paralog ratios (right).
**B.** Average log-expression vs. log-ratio of a representative sample (E17 Rep 4) against all other samples among all individual protein species (left) and protein paralog ratios (right) in the E17 (E) vs. P1 (P) mouse heart experiments.

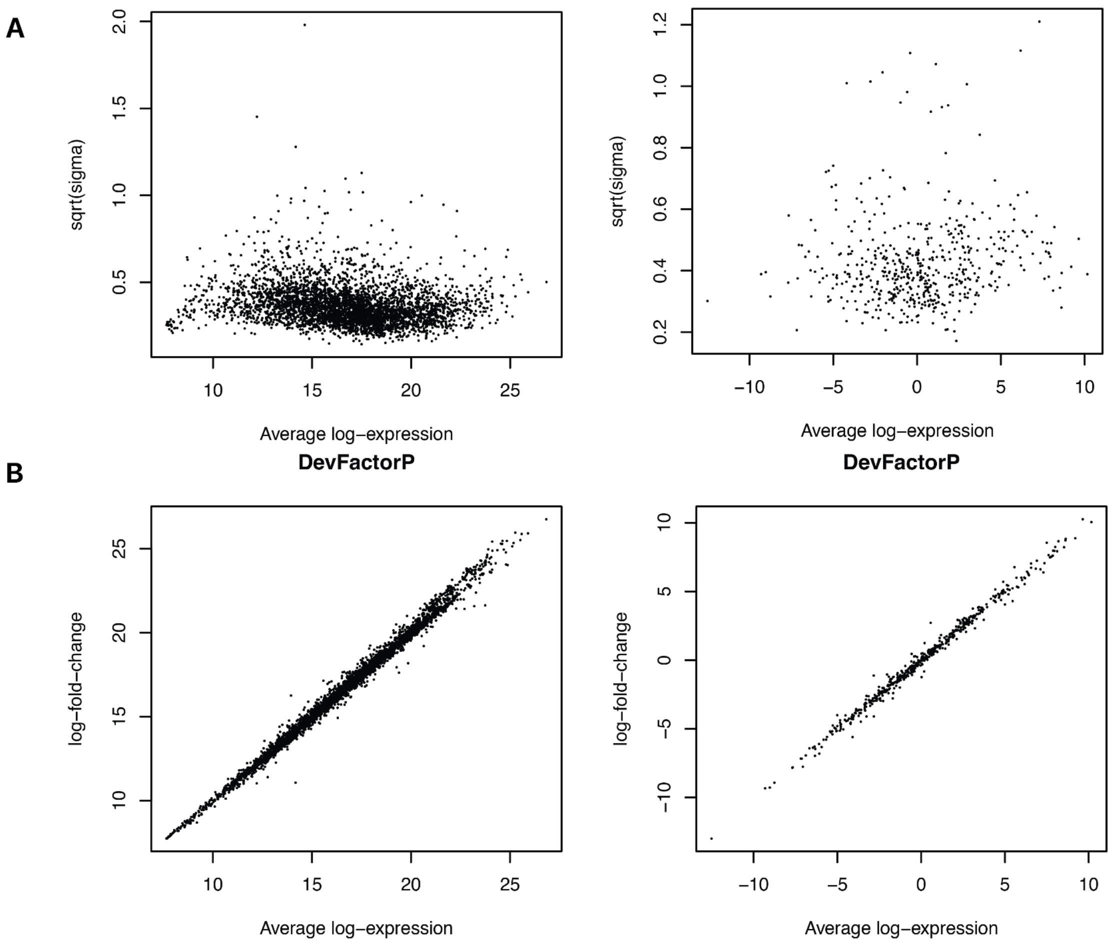


**Figure S2. Comparison of limma diagnostic plots in individual protein species and ratio analysis (continued).**

**A.** Average log-expression vs. fitting residual variance (Sigma) after linear model fitting, among all individual protein species (left) and protein paralog ratios (right) in the E17 (E) vs. P1 (P) mouse heart experiments.

**B.** Log expression over fold-change plot for developmental stage factor (perinatal) between all individual protein species (left) and protein paralog ratios (right).

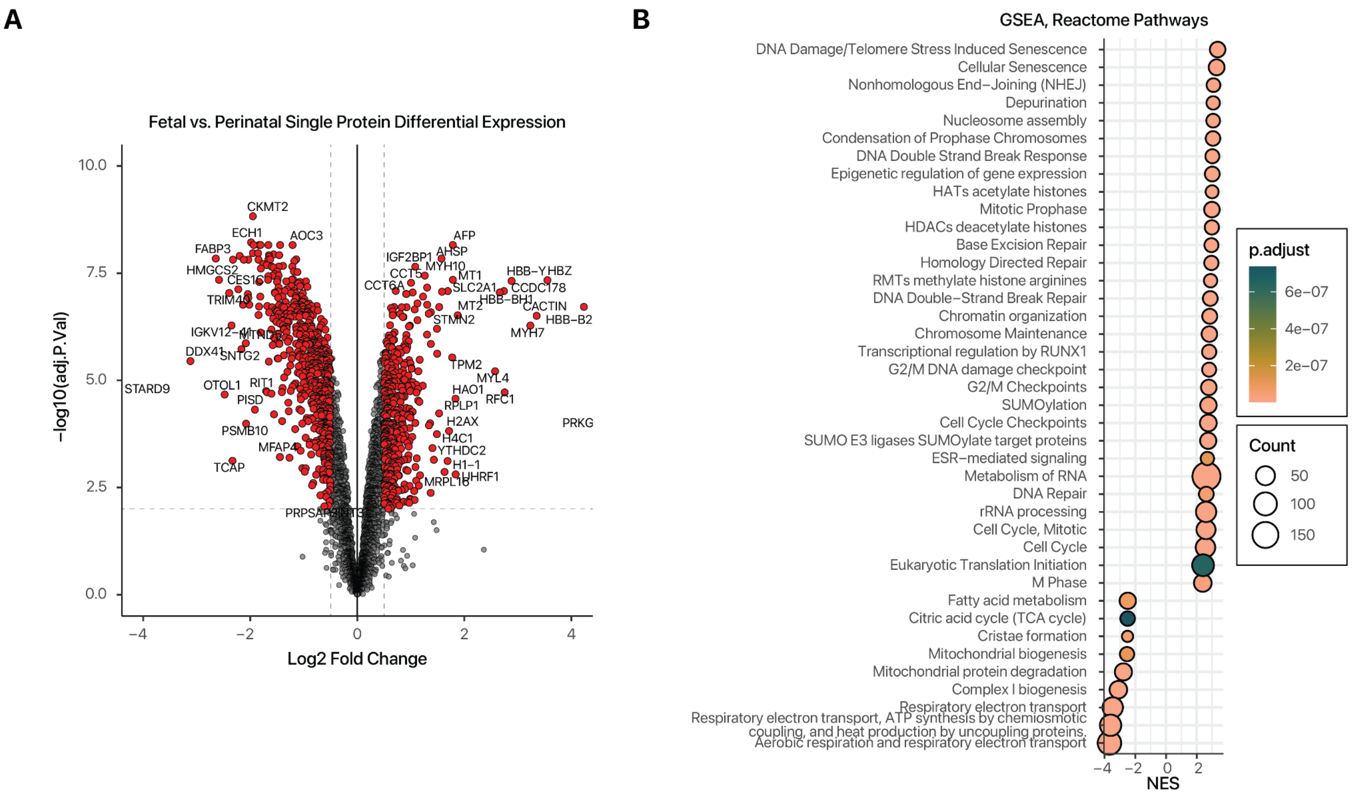


**Figure S3. Individual protein abundance changes in fetal vs. perinatal hearts.**

**A.** Volcano plot showing the log2 fold-change (x-axis) vs. –log10 P value (y-axis) of compared individual proteins in E17 vs. P1 hearts.

**B.** Gene set enrichment analysis (GSEA) of protein fold-change (E17/P1) against Reactome pathways. Color: GSEA adjusted P values; x-axis: GSEA normalized enrichment score (NES); size: number of proteins mapped to pathway.

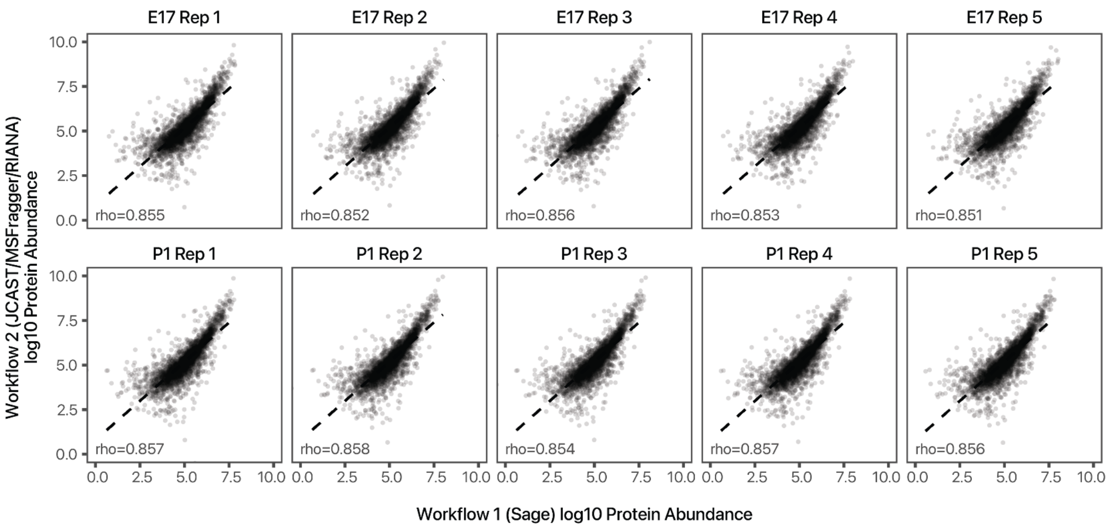


**Figure S4. Comparison of protein quantification workflows**
Scatter plots showing the measured protein abundance (MS1 and MS2 combined) in (x-axis) the Sage/Swiss-Prot workflow and (y-axis) the JCAST/MSFragger workflow. Robust correlation (Spearman's correlation coefficient rho: ~0.85) was observed across ten samples over 5 orders of magnitude in protein abundance in the fetal/postnatal comparisons between the two workflows, despite the use of different search engine, post-processing step, MS1 peak area integration, and TMT intensity extraction tools.

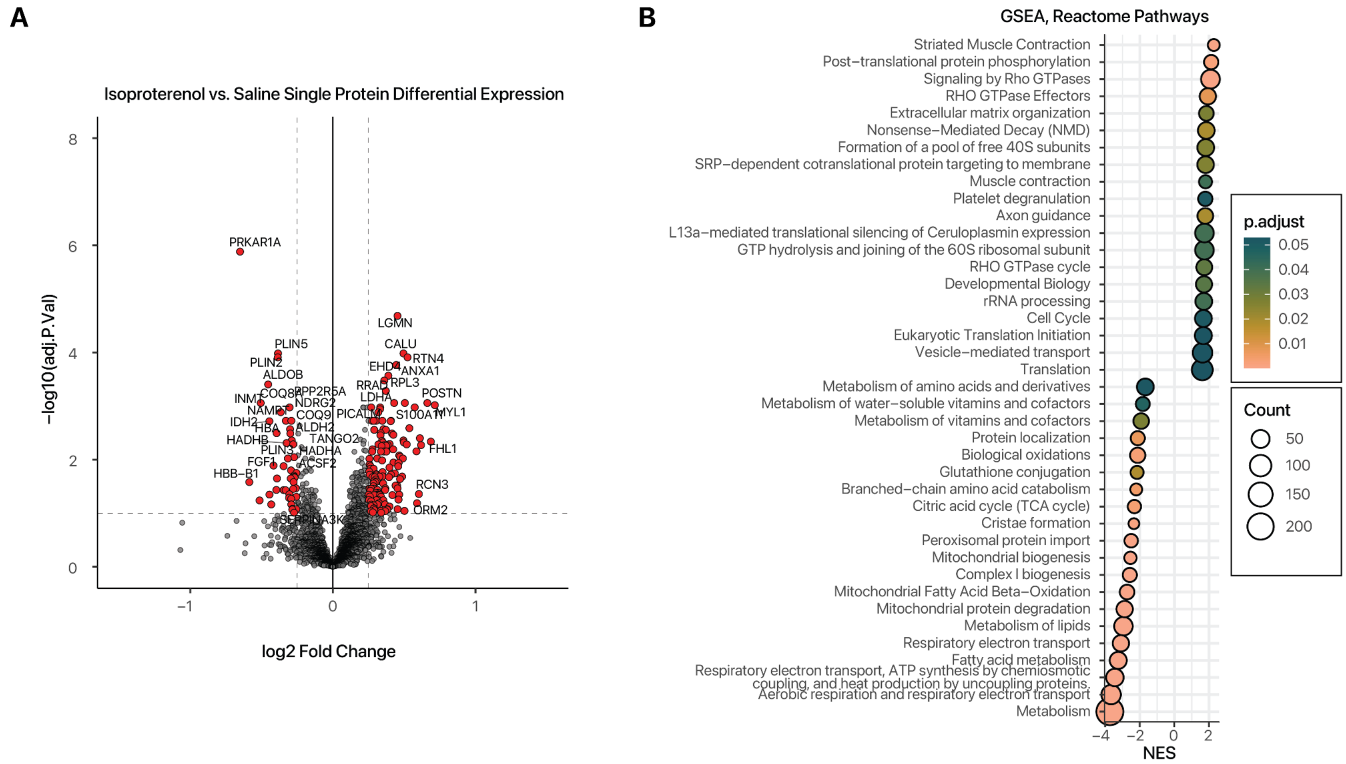


**Figure S5. Individual protein abundance changes in hypertrophy vs. normal hearts.**
**A.** Volcano plot showing the log2 fold-change (x-axis) vs. –log10 P value (y-axis) of compared individual proteins (hypertrophy/normal).
**B.** Gene set enrichment analysis (GSEA) of protein fold-change (hypertrophy/normal) against Reactome pathways. Color: GSEA adjusted P values; x-axis: GSEA normalized enrichment score (NES); size: number of proteins mapped to pathway.

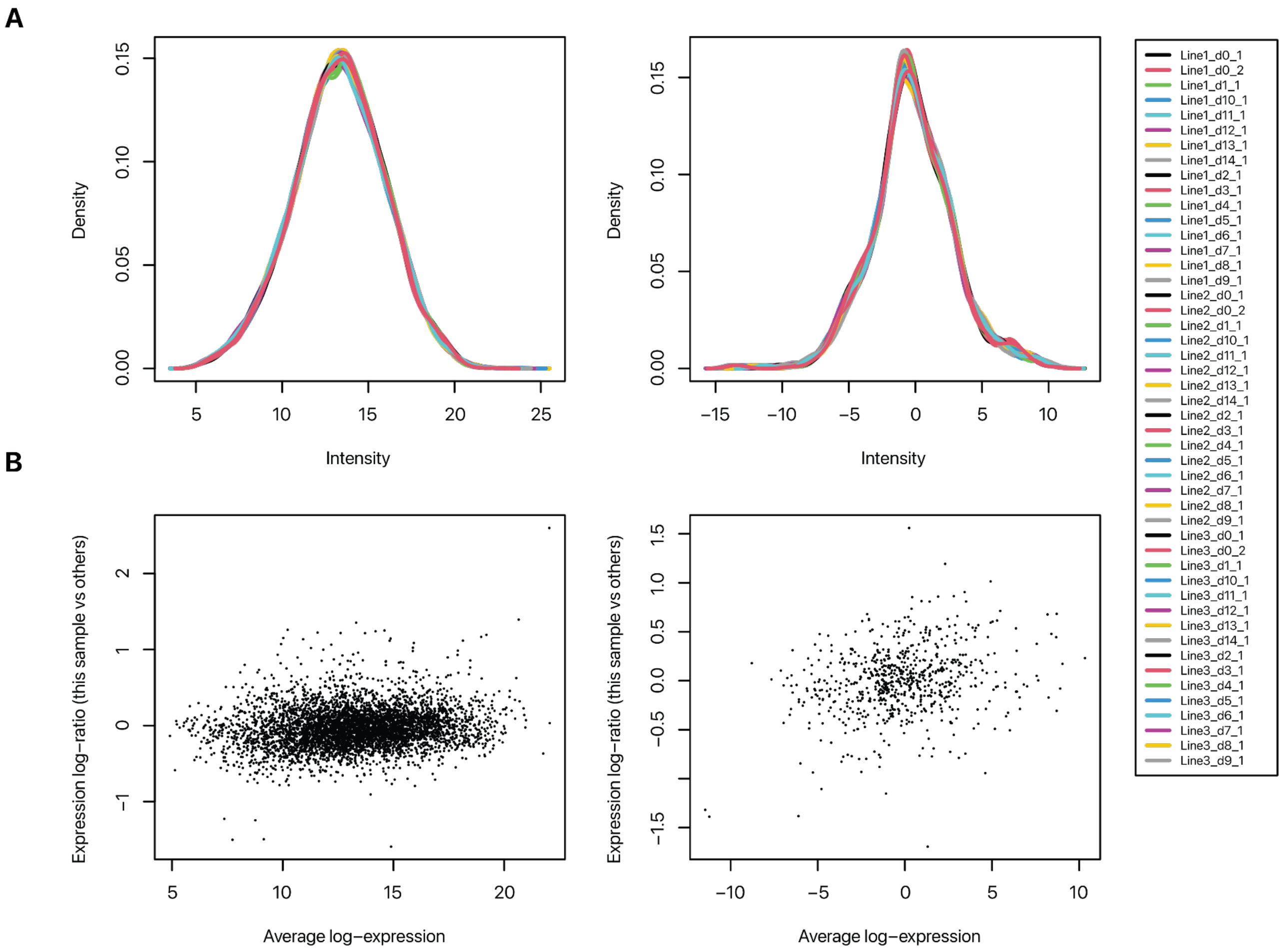


**Figure S6. hiPSC limma diagnostic plots.**
**A.** Distribution of generalized log intensity among all individual protein species (left) and protein paralog ratios (right).
**B.** Average log-expression vs. log-ratio of a representative sample (Line1_d10_1) against all other samples among all individual protein species (left) and protein paralog ratios (right) in the hiPSC differentiation data set.

A

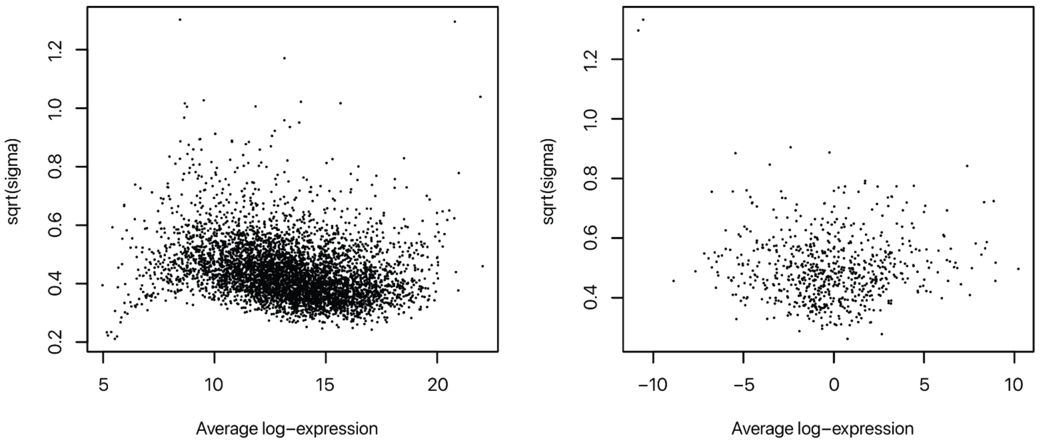


B

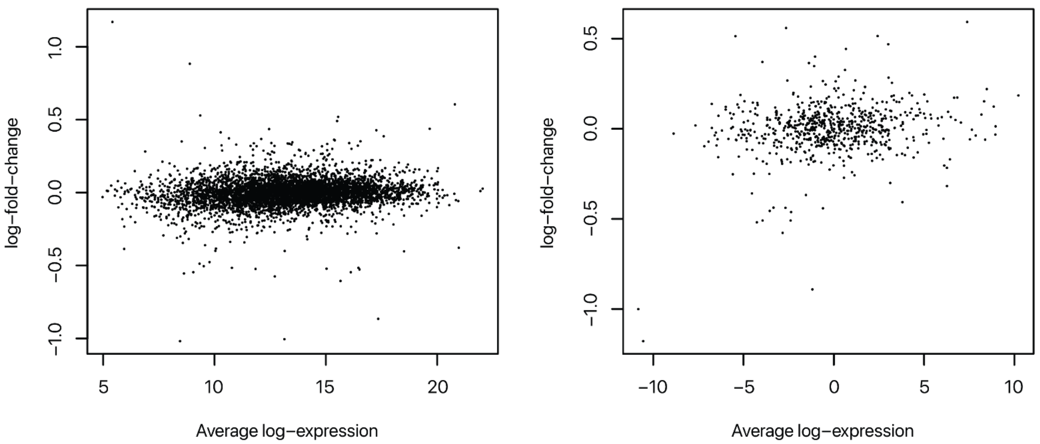


**Figure S7. hiPSC limma diagnostic plots (continued).**

**A.** Average log-expression vs. fitting residual variance (Sigma) after linear model fitting, among all individual protein species (left) and protein paralog ratios (right) in the hiPSC differentiation data set.

**B.** Log expression over fold-change plot for line factor (line 3) between all individual protein species (left) and protein paralog ratios (right).

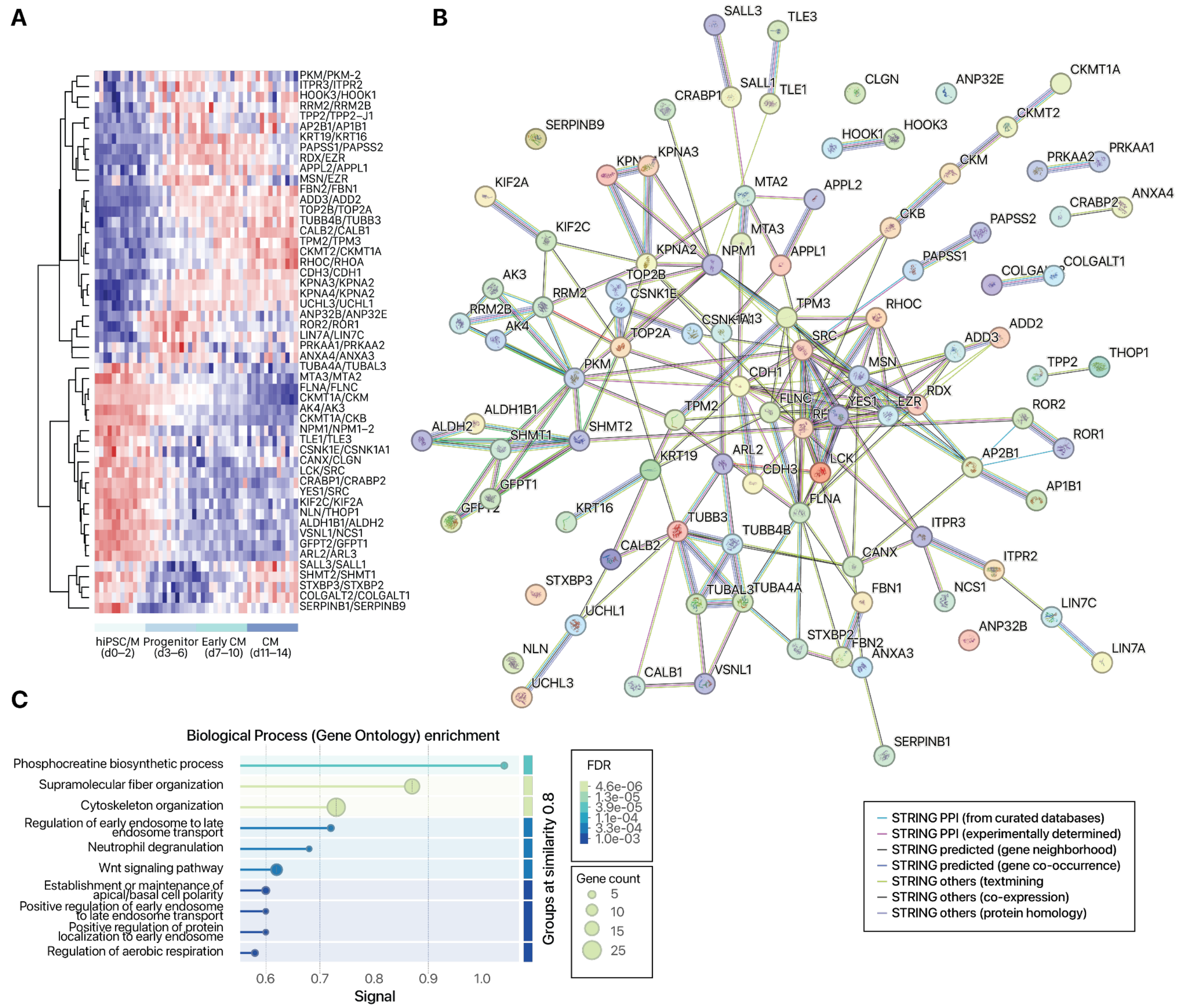

**Figure S8. Differential protein isoform usage in hiPSC/mesoderm to cardiac progenitor transition.**
**A.** Heatmap showing isoform pairs with significantly different usage in hiPSC/mesoderm to progenitor transition. Colors: row standardized ratios.
**B.** STRING network graph of proteins involved in differential isoform usage. Edge colors: STRING interaction type.
**C.** STRING enrichment graph of proteins involved in differential isoform usage. Colors: FDR.

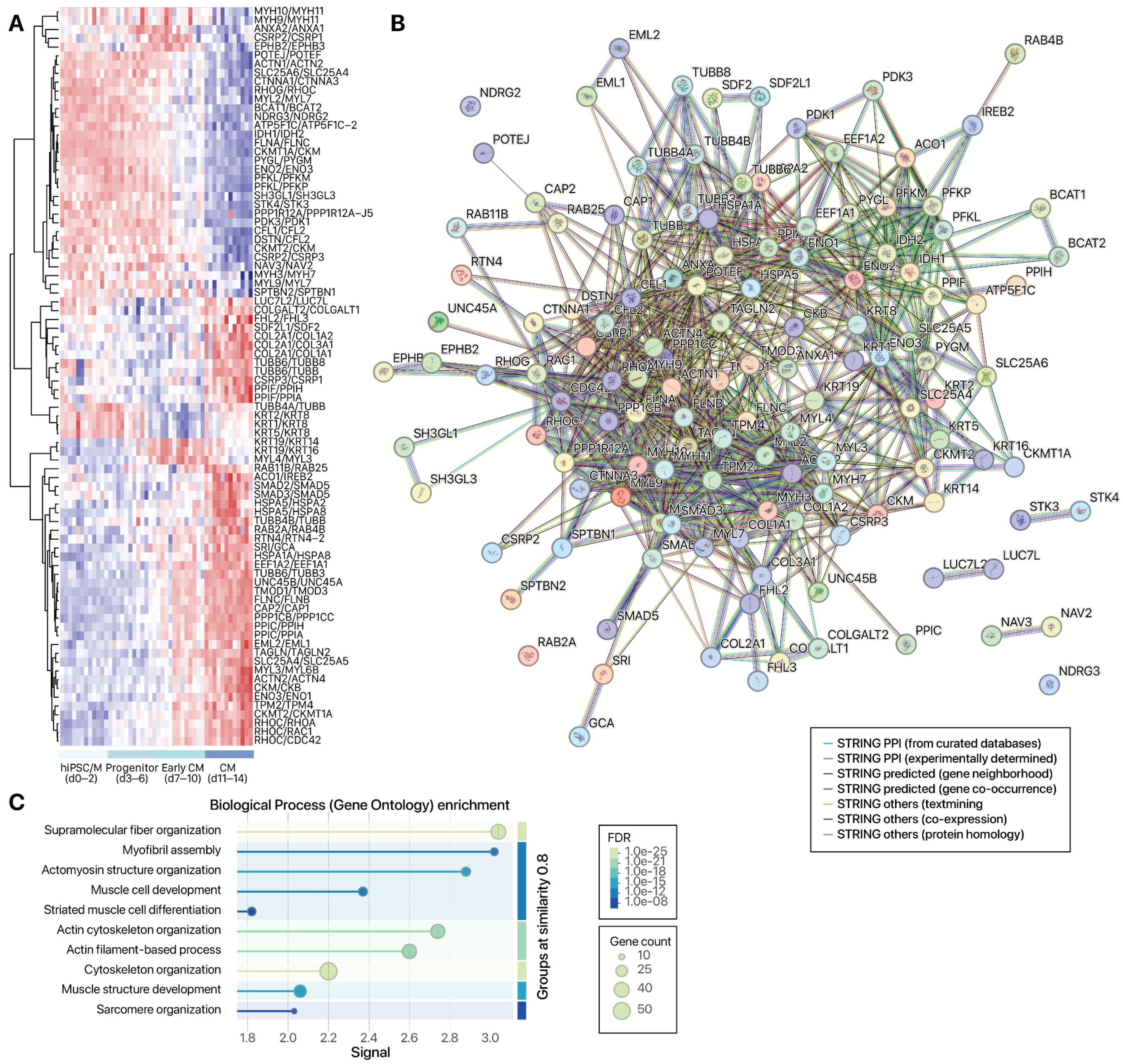


**Figure S9. Differential protein isoform usage in early CM to CM transition.**

**A.** Heatmap showing isoform pairs with significantly different usage in early CM to CM transition. Colors: row standardized ratios.

**B.** STRING network graph of proteins involved in differential isoform usage. Edge colors: STRING interaction type.

**C.** STRING enrichment graph of proteins involved in differential isoform usage. Colors: FDR.

**References in Supplemental Material**

1. Zecha J, et al. TMT Labeling for the Masses: A Robust and Cost-efficient, In-solution Labeling Approach. *Mol Cell Proteomics MCP*. 2019;18(7):1468–1478.

2. Ahrné E, et al. Exploiting the multiplexing capabilities of tandem mass tags for high-throughput estimation of cellular protein abundances by mass spectrometry. *Methods San Diego Calif*. 2015;85:100–107.

3. Clark DJ, et al. Integrated Proteogenomic Characterization of Clear Cell Renal Cell Carcinoma. *Cell*. 2019;179(4):964-983.e31.

4. Wang H, et al. Tissue-based absolute quantification using large-scale TMT and LFQ experiments. *PROTEOMICS*. 2023;23(20):2300188.

5. Wang J, et al. Pan-Cancer Proteomics Analysis to Identify Tumor-Enriched and Highly Expressed Cell Surface Antigens as Potential Targets for Cancer Therapeutics. *Mol Cell Proteomics*. 2023;22(9):100626.

6. Lam MPY, et al. Protein kinetic signatures of the remodeling heart following isoproterenol stimulation. *J Clin Invest*. 2014;124(4):1734–1744.

7. Han Y, et al. Proteogenomics reveals sex-biased aging genes and coordinated splicing in cardiac aging. *Am J Physiol-Heart Circ Physiol*. 2022;323(3):H538–H558.

8. Lazear MR. Sage: An Open-Source Tool for Fast Proteomics Searching and Quantification at Scale. *J Proteome Res*. 2023;22(11):3652–3659.

9. The UniProt Consortium, et al. UniProt: the Universal Protein Knowledgebase in 2023. *Nucleic Acids Res*. 2023;51(D1):D523–D531.

10. Lau E, et al. Splice-Junction-Based Mapping of Alternative Isoforms in the Human Proteome. *Cell Rep*. 2019;29(11):3751-3765.e5.

11. Searle BC, Yergey AL. An efficient solution for resolving iTRAQ and TMT channel cross-talk. *J Mass Spectrom*. 2020;55(8):e4354.

12. Dostal V, et al. Proteomic signatures of acute oxidative stress response to paraquat in the mouse heart. *Sci Rep*. 2020;10(1):18440.

13. Savitski MM, et al. A Scalable Approach for Protein False Discovery Rate Estimation in Large Proteomic Data Sets. *Mol Cell Proteomics MCP*. 2015;14(9):2394–2404.

14. Currie J, et al. Simultaneous proteome localization and turnover analysis reveals spatiotemporal features of protein homeostasis disruptions. *Nat Commun*. 2024;15(1):2207.

15. Schwanhäusser B, et al. Global quantification of mammalian gene expression control. *Nature*. 2011;473(7347):337–342.

16. Huang Q, et al. PaxDb 5.0: Curated Protein Quantification Data Suggests Adaptive Proteome Changes in Yeasts. *Mol Cell Proteomics*. 2023;22(10):100640.

17. Geiger T, et al. Initial quantitative proteomic map of 28 mouse tissues using the SILAC mouse. *Mol Cell Proteomics MCP*. 2013;12(6):1709–1722.

18. Huttlin EL, et al. A tissue-specific atlas of mouse protein phosphorylation and expression. *Cell*. 2010;143(7):1174–1189.

19. Lau E, et al. A large dataset of protein dynamics in the mammalian heart proteome. *Sci Data*. 2016;3:160015.

20. Martin FJ, et al. Ensembl 2023. *Nucleic Acids Res*. 2023;51(D1):D933–D941.

21. Durinck S, et al. Mapping identifiers for the integration of genomic datasets with the R/Bioconductor package biomaRt. *Nat Protoc*. 2009;4(8):1184–1191.

22. Han Y, et al. Determining Alternative Protein Isoform Expression Using RNA Sequencing and Mass

Spectrometry. *STAR Protoc*. 2020;1(3):100138.

23. Ludwig RW, Lau E. JCAST: Sample-specific protein isoform databases for mass spectrometry-based proteomics experiments. *Softw Impacts*. 2021;10:100163.

24. Kong AT, et al. MSFragger: ultrafast and comprehensive peptide identification in mass spectrometry-based proteomics. *Nat Methods*. 2017;14(5):513–520.

25. Yang KL, et al. MSBooster: improving peptide identification rates using deep learning-based features. *Nat Commun*. 2023;14(1):4539.

26. The M, et al. Fast and Accurate Protein False Discovery Rates on Large-Scale Proteomics Data Sets with Percolator 3.0. *J Am Soc Mass Spectrom*. 2016;27(11):1719–1727.

27. Hammond DE, et al. Harmonizing Labeling and Analytical Strategies to Obtain Protein Turnover Rates in Intact Adult Animals. *Mol Cell Proteomics*. 2022;21(7):100252.

28. Gillespie M, et al. The reactome pathway knowledgebase 2022. *Nucleic Acids Res*. 2022;50(D1):D687–D692.

29. Yu G, He Q-Y. ReactomePA: an R/Bioconductor package for reactome pathway analysis and visualization. *Mol Biosyst*. 2016;12(2):477–479.

30. Szklarczyk D, et al. The STRING database in 2023: protein–protein association networks and functional enrichment analyses for any sequenced genome of interest. *Nucleic Acids Res*. 2023;51(D1):D638–D646.

31. Frankish A, et al. GENCODE 2021. *Nucleic Acids Res*. 2021;49(D1):D916–D923.

32. Dobin A, et al. STAR: ultrafast universal RNA-seq aligner. *Bioinforma Oxf Engl*. 2013;29(1):15–21.

33. Pertea M, et al. StringTie enables improved reconstruction of a transcriptome from RNA-seq reads. *Nat Biotechnol*. 2015;33(3):290–295.

34. Love MI, Huber W, Anders S. Moderated estimation of fold change and dispersion for RNA-seq data with DESeq2. *Genome Biol*. 2014;15(12):550.

35. Zhu A, Ibrahim JG, Love MI. Heavy-tailed prior distributions for sequence count data: removing the noise and preserving large differences. *Bioinforma Oxf Engl*. 2019;35(12):2084–2092.
